# CSF p-tau205: a biomarker of tau pathology in Alzheimer’s disease

**DOI:** 10.1007/s00401-023-02659-w

**Published:** 2024-01-06

**Authors:** Juan Lantero-Rodriguez, Laia Montoliu-Gaya, Andrea L. Benedet, Agathe Vrillon, Julien Dumurgier, Emmanuel Cognat, Wagner S. Brum, Nesrine Rahmouni, Jenna Stevenson, Stijn Servaes, Joseph Therriault, Bruno Becker, Gunnar Brinkmalm, Anniina Snellman, Hanna Huber, Hlin Kvartsberg, Nicholas J. Ashton, Henrik Zetterberg, Claire Paquet, Pedro Rosa-Neto, Kaj Blennow

**Affiliations:** 1 Department of Psychiatry and Neurochemistry, Institute of Neuroscience and Physiology, The Sahlgrenska Academy at the University of Gothenburg, Mölndal, Sweden; 2 Cognitive Neurology Center, Université de Paris Cité, GHU Nord APHP Hospital Lariboisière Fernand Widal, Paris, France; 3 Graduate Program in Biological Sciences: Biochemistry, Universidade Federal Do Rio Grande Do Sul (UFRGS), Porto Alegre, Brazil; 4 Montreal Neurological Institute, Montreal, QC Canada; 5 Department of Neurology and Neurosurgery, McGill University, Montreal, QC Canada; 6 Turku PET Centre, University of Turku, Turku University Hospital, Turku, Finland; 7 Clinical Neurochemistry Laboratory, Sahlgrenska University Hospital, Mölndal, Sweden; 8 Wallenberg Centre for Molecular and Translational Medicine, University of Gothenburg, Gothenburg, Sweden; 9 Department of Old Age Psychiatry, Maurice Wohl Clinical Neuroscience Institute, King’s College London, London, UK; 10 NIHR Biomedical Research Centre for Mental Health and Biomedical Research Unit for Dementia at South London and Maudsley NHS Foundation, London, UK; 11 Department of Neurodegenerative Disease, Queen Square Institute of Neurology, University College London, London, UK; 12 UK Dementia Research Institute, University College London, London, UK; 13 Hong Kong Center for Neurodegenerative Diseases, Hong Kong, China; 14 Wisconsin Alzheimer’s Disease Research Center, School of Medicine and Public Health, University of Wisconsin, University of Wisconsin-Madison, Madison, WI USA

**Keywords:** CSF, p-tau205, Alzheimer’s disease, Tau, Biomarker

## Abstract

Post-mortem staging of Alzheimer’s disease (AD) neurofibrillary pathology is commonly performed by immunohistochemistry using AT8 antibody for phosphorylated tau (p-tau) at positions 202/205. Thus, quantification of p-tau205 and p-tau202 in cerebrospinal fluid (CSF) should be more reflective of neurofibrillary tangles in AD than other p-tau epitopes. We developed two novel Simoa immunoassays for CSF p-tau205 and p-tau202 and measured these phosphorylations in three independent cohorts encompassing the AD *continuum*, non-AD cases and cognitively unimpaired participants: a discovery cohort (*n* = 47), an unselected clinical cohort (*n* = 212) and a research cohort well-characterized by fluid and imaging biomarkers (*n* = 262). CSF p-tau205 increased progressively across the AD *continuum,* while CSF p-tau202 was increased only in AD and amyloid (Aβ) and tau pathology positive (A+T+) cases (*P* < 0.01). In A+ cases, CSF p-tau205 and p-tau202 showed stronger associations with tau-PET (*r*_Sp205_ = 0.67, *r*_Sp202_ = 0.45) than Aβ-PET (*r*_Sp205_ = 0.40, *r*_Sp202_ = 0.09). CSF p-tau205 increased gradually across tau-PET Braak stages (*P* < 0.01), whereas p-tau202 only increased in Braak V–VI (*P* < 0.0001). Both showed stronger regional associations with tau-PET than with Aβ-PET, and CSF p-tau205 was significantly associated with Braak V–VI tau-PET regions. When assessing the contribution of Aβ and tau pathologies (indexed by PET) to CSF p-tau205 and p-tau202 variance, tau pathology was found to be the most prominent contributor in both cases (CSF p-tau205: *R*^2^ = 69.7%; CSF p-tau202: *R*^2^ = 85.6%) Both biomarkers associated with brain atrophy measurements globally (*r*_Sp205_ = − 0.36, *r*_Sp202_ = − 0.33) and regionally, and correlated with cognition (*r*_Sp205_ = − 0.38/− 0.40, *r*_Sp202_ = − 0.20/− 0.29). In conclusion, we report the first high-throughput CSF p-tau205 immunoassay for the in vivo quantification of tau pathology in AD, and a potentially cost-effective alternative to tau-PET in clinical settings and clinical trials.

**Supplementary Information:**

The online version contains supplementary material available at 10.1007/s00401-023-02659-w.

## Introduction

Neuropathological confirmation of amyloid-β (Aβ) plaques and tau neurofibrillary tangles (NFTs) remains the gold standard for definitive diagnosis of Alzheimer’s disease (AD) [10]. Aβ plaques are the result of the aberrant accumulation of Aβ peptides in the extracellular space, while NFTs are constituted by intraneuronal fibrils of highly phosphorylated tau protein. The spatiotemporal spreading pattern of Aβ plaques and NFTs in brain are referred to as Thal and Braak stages, respectively [11, 24]. Interestingly, unlike Thal Aβ stages, Braak stages have been shown to correlate with disease progression and cognitive decline [35, 43]. At post-mortem examination, Braak stages are commonly determined by immunostaining using AT8, an antibody obtained by immunizing mice with paired helical filament or PHF-tau [28], and which epitope is centred on phosphorylated tau (p-tau) at serine 202 and threonine 205 [12, 14, 23, 40]. The ability of this antibody to stain pathological tau aggregates extends far beyond AD, and therefore, these two tau phosphorylations (p-tau202 and p-tau205) have been traditionally associated with tau pathology in the brain. However, while great progress has been achieved in measuring several soluble p-tau species capable of tracking AD pathology in vivo [1], p-tau205 and p-tau202 remain comparatively under-explored as viable fluid biomarkers.

In recent years, the development of ultra-sensitive immunoassays [1, 17, 20, 45] and mass spectrometry (MS) [4, 6, 13] methods have greatly expanded the knowledge regarding p-tau species in biofluids, and most importantly, highlighted their potential value as biomarkers for AD. Several studies have shown that most p-tau variants (e.g., p-tau181, p-tau217, p-tau231 and p-tau235) share common features: all are highly specific for AD, levels in CSF increase during preclinical AD, and associate well with in vivo Aβ and to a lesser degree, tau pathology [1, 19, 20, 22, 45]. However, they do present some dissimilarities. For example, p-tau217 has been shown to display the largest fold-change in the symptomatic phase of AD, and various reports indicate it may provide the best performance for AD diagnosis and disease monitoring [2, 3, 17, 30]. P-tau231 has been demonstrated to be the earliest p-tau biomarker to abnormally emerge during preclinical AD stages, even prior to detectable Aβ pathology by positron emission tomography (PET) [1, 30, 45]. P-tau235 has been proposed as a biomarker capable of staging preclinical AD, due to its involvement in a sequential phosphorylation event observed in neuropathology confirmed brain tissue and CSF [20]. A study using MS demonstrated that tau phosphorylation in AD is a dynamic process, with each site-specific tau phosphorylation abnormally emerging at different time points along the course of the disease. While p-tau217 and p-tau181 showed initial early increases in parallel to Aβ plaque formation and prior to neuronal dysfunction, p-tau205 showed a late increase closer to atrophy, hypometabolism, and symptom onset [5]. In a later publication, increased CSF p-tau205, but not other phosphorylated tau species, correlated with lower white matter integrity [44]. In addition, a recent study showed that CSF p-tau205 did not strongly associate with Aβ-PET but was among the best predictors of tau-PET status [7]. Altogether, these results suggest that p-tau205 might pose biomarker potential detecting mid-to-late stages of AD progression and tracking tau pathology. On the other hand, literature on CSF p-tau202 is somewhat contradictory. Some studies have indicated that CSF p-tau202 is inversely associated with Aβ-PET, and the phosphorylation rate decreases with disease severity [6, 8], whereas others have shown increases along the AD *continuum* and positive correlations with Aβ and tau-PET [13].

All previous works reporting the levels of CSF p-tau205 and p-tau202 have used MS methods for quantification which have limited application compared with immunoassay for wide-scale research and clinical use. In this study, we report the development of the two first ultrasensitive immunoassays specifically measuring p-tau205 and p-tau202, key markers of neuropathological tau in AD, and assess their ability to reflect in vivo neurofibrillary pathology in AD. Furthermore, we investigate the diagnostic performance of both biomarkers in clinical settings and thoroughly characterize the link between these two CSF p-tau species with in vivo measurements of Aβ pathology, tau pathology, neurodegeneration, and cognition.

## Materials and methods

### Sample cohorts

The biomarker potential of the in-house-developed CSF p-tau202 and p-tau205 assays was assessed in three independent cohorts:

### Discovery cohort

The discovery cohort was comprised of patients with biologically defined AD (*n* = 21) and neurological controls (*n* = 26) (Supplementary Table 1) clinically assessed in the Sahlgrenska University Hospital, Gothenburg, Sweden. AD cases were admitted for clinical evaluation for suspected AD, underwent lumbar puncture, and core AD CSF biomarkers were measured. Patients classified as AD displayed the typical AD CSF biomarker profile (CSF Aβ42 < 530 ng/L, p-tau181 > 60 ng/L, t-tau > 350 ng/L, all measured using INNOTEST ELISA). Neurological controls included patients with cognitive complains but no CSF biomarkers abnormalities. Individuals with other neurological disorders or with concomitant inflammatory diseases were not included.

### Paris cohort

The Paris cohort is a memory clinic real world cohort including a total of 212 subjects who underwent clinical assessments and core CSF AD biomarker analysis at the Centre of Cognitive Neurology at Lariboisière Fernand-Widal Hospital, Université de Paris Cité. All patients underwent a detailed clinical evaluation including both personal and family histories, extensive neurological and neuropsychological assessment, and CSF collection. Clinical diagnosis was made by specialists in multidisciplinary consensus meetings, taking into account Lumipulse CSF biomarkers results and the validated criteria for the clinical diagnostic of AD dementia [15], AD-MCI [26, 38], dementia with Lewy bodies (DLB) [26] and frontotemporal dementia (FTD) [41]. AD patients exhibited abnormalities for core CSF biomarkers on the AD *continuum* [15]. MCI of other causes (non-AD MCI) included subjects with psychiatric disorder, systemic disease, or sleep apnea. Non-AD MCI showed normal core CSF biomarkers profile or suspected non-Alzheimer pathophysiology (normal Aβ42/401-42/40, high p-tau and/or high t-tau). Non-AD dementia cases comprised patients with cognitive decline related to DLB, FTD, vascular cognitive impairment and dementia (VCID), and Creutzfeldt-Jakob disease (CJD). Subjective cognitive decline patients included individuals with several years of clinical follow-up for clinical complains but presenting with normal cognitive testing and no abnormalities at CSF and imaging examinations [18, 31]. Participants were classified according to the clinical syndrome (cognitively unimpaired [CU], mild cognitively impaired [MCI] or dementia) and the CSF Aβ status (A−/A+, as defined by Lumipulse CSF Aβ42/40) into: CU− (*n* = 21), MCI− (*n* = 39), MCI+ (*n* = 48), AD (all cases were Aβ+ , *n* = 73), nonAD- (non-AD dementia Aβ-, *n* = 25) and nonAD+ (non-AD dementia Aβ+ , *n* = 6) (Table 1). Participants were also stratified based on Aβ (A) and tau (T) status defined using Lumipulse CSF Aβ42/40 and p-tau181, respectively, into A−T− (*n* = 82), A+T− (*n* = 27) and A+T+ (*n* = 99) (A−T+ [*n* = 4] cases were considered suspected non-AD pathology [SNAP], and were therefore not included in the statistical analysis of the AT groups, but they are depicted in the AT boxplots) (Supplementary Table 2). Lumipulse cut-offs for Paris cohort are published elsewhere [21].

**Table 1:** Demographics of the Paris and TRIAD CSF cohorts

Paris cohort (*n* = 212)	–	CU− (*n* = 21)	MCI− (*n* = 39)	NonAD− (*n* = 25)	–	MCI+ (*n* = 48)	NonAD+ (*n* = 6)	AD (*n* = 73)	*P* value
Age	**–**	64.38 (9.50)	66.87 (9.87)	65.96 (8.04)	**–**	71.85 (7.74)	67.33 (7.09)	71.97 (8.43)	< 0.001
Males (%)	**–**	14 (66.7)	24 (61.5)	11 (0.44)	**–**	31 (64.6)	3 (50)	46 (63.0)	ns
*APOE* ε4 carriers (%)	**–**	6/21 (28.6)	5/24 (20.8)	5/6 (83.3)	**–**	27/48 (56.3)	4/39 (10.3)	47/72 (65.3)	< 0.001
MMSE score (available cases)	**–**	20	37	25	**–**	47	6	72	
MMSE score	**–**	27.15 (2.52)	24.22 (3.87)	24.12 (4.95)	**–**	23.62 (4.54)	18.33 (6.62)	19.08 (5.63)	< 0.001
Lumipulse CSF (pg/mL)									
Aβ42/40	**–**	0.09 (0.01)	0.09 (0.01)	0.09 (0.01)	**–**	0.05 (0.01)	0.06 (0.005)	0.042 (0.01)	< 0.001
p-tau181	**–**	32.81 (8.64)	36.73 (15.15)	32.74 (10.26)	**–**	86.22 (47.55)	51.93 (16.90)	115.62 (59.85)	< 0.001
t-tau	**–**	243.10 (70.88)	301.28 (140.85)	364.24 (355.66)	**–**	565.52 (279.29)	365.17 (133.30)	736.05 (394.52)	< 0.001
GU CSF biomarkers (pg/mL)									
p-tau202	**–**	1.93 (0.96)	2.41 (1.45)	2.09 (1.53)	**–**	3.07 (1.64)	2.34 (2.52)	3.66 (1.67)	< 0.001
p-tau205	**–**	1.56 (0.30)	1.94 (1.41)	1.59 (0.60)	**–**	3.83 (1.86)	2.72 (1.13)	5.33 (2.69)	< 0.001

TRIAD cohort (*n* = 262)	Young (*n* = 27)	CU− (*n* = 74)	MCI− (*n* = 17)	NonAD− (*n* = 28)	CU+ (*n* = 33)	MCI+ (*n* = 35)	NonAD+ (*n* = 5)	AD (*n* = 43)	*P *value
Age, years	23.0 (1.9)	68.8 (9.8)	68.2 (11.6)	62.2 (11.4)	70.4 (7.0)	71.3 (6.0)	71.0 (6.2)	65.2 (8.0)	< 0.001
Males (%)	11 (40.7)	30 (40.5)	9 (52.9)	11 (39.3)	13 (39.4)	15 (42.9)	2 (40.0)	21 (48.8)	ns
Formal education, years	16.7 (1.5)	15.6 (4.3)	12.6 (5.9)	12.3 (6.0)	14.2 (3.4)	15.5 (4.2)	5.8 (8.3)	12.3 (6.2)	< 0.001
*APOE* ε4 carriers (%)	6/27 (22.2)	17/74 (23.0)	2/15 (13.3)	3/21 (14.3)	13/33 (39.4)	20/32 (62.5)	2/4 (50.0)	22/35 (62.9)	< 0.001
MMSE score (available cases)	27	69	14	15	31	29	2	31	
MMSE score	29.78(0.51)	29.20(1.02)	28.14(1.56)	25.60(5.99)	29.13(0.88)	28.10(1.99)	24.50(2.12)	20.64(6.13)	< 0.001
Lumipulse CSF (pg/mL)									
Aβ42/40	0.09 (0.01)	0.09 (0.01)	0.09 (0.01)	0.09 (0.01)	0.05 (0.01)	0.05 (0.01)	0.05 (0.01)	0.04 (0.01)	< 0.001
p-tau181	22.5 (7.1)	33.9 (10.8)	40.2 (12.0)	31.2 (12.7)	58.3 (32.4)	79.5 (38.0)	64.4 (22.6)	126.7 (79.5)	< 0.001
t-tau	195.4 (47.6)	292.7 (112.7)	320.4 (89.0)	307.4 (156.2)	419.9 (184.9)	504.8 (208.7)	620.0 (398.5)	839.8 (456.2)	< 0.001
Aβ-PET (available cases)	27	67	14	20	32	34	1	32	
SUVR	1.21 (0.07)	1.29 (0.12)	1.37 (0.16)	1.26 (0.22)	1.83 (0.48)	2.33 (0.55)	-	2.30 (0.51)	< 0.001
Tau-PET (available cases)	26	67	14	19	31	33	1	32	
SUVR	0.84(0.08)	0.83 (0.09)	0.81(0.09)	0.82(0.11)	0.95(0.23)	1.33(0.55)	-	2.37 (0.89)	< 0.001
VBM (available cases)	26	64	14	18	30	30	1	30	
mm^3^	0.57(0.06)	0.46(0.05)	0.44(0.04)	0.41(0.07)	0.46(0.05)	0.43(0.06)	-	0.39(0.07)	< 0.001
GU CSF biomarkers (pg/mL)									
p-tau202	1.05 (0.72)	1.81 (1.03)	1.42 (0.65)	2.10 (1.38)	2.21 (1.16)	2.57 (1.24)	2.83 (0.85)	3.26 (1.52)	< 0.001
p-tau205	1.19 (0.37)	1.75 (0.48)	1.90 (0.39)	1.62 (0.53)	2.72 (1.37)	3.85 (1.59)	3.25 (0.72)	5.50 (2.87)	< 0.001

Data are shown as mean (SD) or *n* (%), as appropriate. Kruskal Wallis test was used to compare age between groups and Pearson’s chi-square to compare sex and APOE ε4 frequencies between groups. Years of education, MMSE and biomarkers levels were compared with a one-way ANOVA adjusted by age and sex

Abbreviations: *Aβ42/40* ratio β-amyloid 42 and 40, *AD* Alzheimer’s disease, *CSF* cerebrospinal fluid, *CU* cognitively unimpaired, *GU* Gothenburg University Simoa assay, *MCI* mild cognitive impairment, *MMSE* Mini-Mental State Examination, *NonAD* non-Alzheimer’s disease, *ns* non-significant, *p-tau181* tau phosphorylated at threonine 181, *p-tau202* tau phosphorylated at serine 202, *p-tau205* tau phosphorylated at threonine 205, *PET* positron emission tomography, *SUVR* standardized uptake value ratio, *t-tau* total tau, *VBM* voxel-based morphometry.

### TRIAD cohort

The cross-sectional samples presented here belong to the Translational Biomarkers of Aging and Dementia (TRIAD) cohort (McGill University, Montreal, Canada). Participants from this research cohort were stratified according to the clinical syndrome (CU, MCI or dementia) and the CSF Aβ status (A−/A+, as defined by Lumipulse CSF Aβ42/40; cut-offs are published elsewhere [9]) into: young (*n* = 27), CU− (*n* = 74), CU+ (*n* = 33), MCI− (*n* = 17), MCI+ (*n* = 35), AD (*n* = 43), nonAD+ (*n* = 5) and nonAD− (*n* = 28) (Table 1). Young and CU participants scored 0 on the Clinical Dementia Rating (CDR) scale with no objective cognitive abnormalities. MCI cases had CDR score of 0.5 and presented objective and subjective memory impairment but preserved the ability to perform daily life activities. AD cases were diagnosed according to the National Institute of Aging and the Alzheimer’s Association criteria for probable AD [27]. NonAD participants included cases suspected of non-Alzheimer pathophysiology with diagnosis including with FTD (behavioural or semantic variant), progressive supranuclear palsy (PSP) or primary progressive aphasia (PPA), all with CDR > 0.5 and negative Aβ-PET scan. Core CSF biomarkers were measured in all participants using a Lumipulse platform (G1200, Fujirebio) at the Clinical Neurochemistry Laboratory, Sahlgrenska University Hospital, Mölndal, Sweden. Participants were further stratified based on the Aβ (A) and tau (T) status defined using Lumipulse CSF Aβ42/40 and p-tau181, respectively, into A−T− (*n* = 135), A+T− (*n* = 28) and A+T+ (*n* = 88). A−T+ [*n* = 11] cases are considered SNAP and were therefore not included in the statistical analysis of the AT groups but are depicted in the AT boxplots (Supplementary Table 3). Participants were also stratified into AT groups using PET to define Aβ (A) and tau (T) status (Supplementary Table 4).

### Simoa CSF p-tau202 and p-tau205 measurements

CSF levels of p-tau202 and p-tau205 were quantified in all three cohorts (Discovery, Paris, and TRIAD) with two in-house developed immunoassays using a Simoa HD-X platform (Quanterix) at the Clinical Neurochemistry Laboratory, Sahlgrenska University Hospital, Mölndal, Sweden. Immunoassay development and validation details are described in the Supplementary Methods. In the CSF p-tau202 immunoassay, a rabbit polyclonal antibody selective against phosphorylated tau at serine 202 (immunogen: synthesized human tau peptide around the phosphorylation site of serine202, ThermoFisher Scientific) was used as capture antibody, whereas a biotinylated mouse monoclonal antibody targeting N-terminal tau (Tau12, BioLegend) was used for detection. Similarly, in the CSF p-tau205 immunoassay, a rabbit polyclonal antibody selective against phosphorylated tau at threonine 205 (immunogen: synthesized human tau peptide around the phosphorylation site of threonine205, ThermoFisher Scientific) was used as capture antibody with biotinylated Tau12 used for detection. Eight-point calibration curves in both assays were generated using commercially available recombinant full-length Tau411 in vitro*-*phosphorylated by GSK-3β (SignalChem) and run in duplicates. Prior to the analysis, samples were allowed to thaw at room temperature for 45 min. Thawed CSF samples were vortexed for 30 s at 2000 rpm and diluted using commercially available Tau2.0 assay diluent (Quanterix). All samples were randomized and analysed blinded. Two internal quality controls (iQC), one low and one high, were run in duplicates at the beginning and the end of each plate. Repeatability and intermediate precision were < 15% for both the CSF p-tau202 and p-tau205 assays.

### Imaging analysis (TRIAD cohort)

A subset of 227 and 223 participants included in this study had, respectively, Aβ and tau pathologies indexed by PET imaging (Supplementary Table 5). Demographics from participants with available Aβ and tau PET as well as CSF p-tau181, p-tau217 and p-tau231 are available in Supplementary Table 6. The structural MRI data was available for a subset of 213 participants (Supplementary Table 7) and was acquired on a 3T Siemens Magnetom where high-resolution T1-weighted images were acquired. The SPM12 tool was used for segmentation of T1-weighted images, which were then non-linearly registered to the ADNI template using DARTEL, as previously reported [46]. MRI images were also processed with an optimized Voxel-Based Morphometry (VBM) protocol. The grey matter VBM images provided an estimation of global brain neurodegeneration, which was generated with an AD-signature mask that is a composite of the entorhinal, inferior temporal, middle temporal, and fusiform regions [16].

Aβ-PET ([^18^F]AZD4694) and tau-PET ([^18^F]MK6240) were acquired with a Siemens High Resolution Research Tomograph (Siemens Medical Solutions, Knoxville, TN), respectively, 40–70 and 90–110 min post-tracer injection. Images were co-registered to individual’s T1-weighted MRI scans and processed following published protocols [36, 47]. The global Aβ load was inferred by the average standardized uptake value ratio (SUVR) of the precuneus, cingulate, inferior parietal, medial prefrontal, lateral temporal, and orbitofrontal cortices using the cerebellar grey matter as reference region. Aβ-PET positivity was established as equal or greater than 1.55 SUVR [48]. For tau-PET, the average SUVR in the meta-ROI region was used to estimate a global tau load. Inferior cerebellar grey matter was used as a reference region, and the cutoff for tau positivity was 1.24 SUVR. In addition, in vivo classification of Braak stages was performed as previously described [37].

### Statistical analysis

Analyses were performed on SPSS (v26, IBM, Armonk, NY), unless otherwise specified. Parametric and non-parametric tests were used when appropriate, based on the data distribution. Thus, comparisons between groups were performed with Mann–Whitney *U* test (two categories), and one-way ANCOVA adjusted by age and sex, followed by Bonferroni-corrected post-hoc analysis. Spearman’s rank (*r*_*S*_) tested the correlation between biomarkers. The accuracy of CSF p-tau biomarkers to distinguish binary outcomes was determined using the receiver operating characteristics (ROC) and presented as area under the curve (AUC), which were compared between each other using DeLong test (MedCalc, Ostend, Belgium). Linear regression models tested the association between biomarkers adjusting for age and sex (R Studio v4.0), and the Akaike information criterion (AICc) and r-squared values are reported to evaluate best-fitting models. In addition, mediation analysis was performed using the *mediate* function (*psych*) in R.

Statistical analysis on imaging data was performed using Rminc where linear regression models were applied voxel-wise to evaluate the association between CSF and imaging biomarkers adjusting by age and sex in all participants or within groups, as described in the results. Adjusted *R*^2^ and t-parametric maps are presented. Random-field theory [50] was applied on the t-parametric maps to correct for multiple comparisons.

### Data availability

Bulk anonymized data can be shared by request from qualified investigators, providing data transfer is in agreement with EU legislation and decisions by the institutional review board of each participating research centre.

### Ethics approval and consent

All participants or their legal relatives in case of severe dementia gave written informed consent to their participation in this study. Collection and analysis of samples were approved by the Ethics Committee at the University of Gothenburg (EPN 140811), the ethic committee of Bichat University, Paris, France (CEERB GHU Nord n°10-037) for Paris cohort, and by the Research Ethics Board of the Montreal Neurological Institute as well as the Faculty of Medicine Research Ethics Office, McGill University.

## Results

### Participant characteristics

In total, we included 521 participants: 47 individuals in the discovery cohort, 212 from a clinical cohort (Paris cohort) and 262 from a research cohort (TRIAD cohort). There were no significant differences in age across groups in the discovery cohort. Significant differences in age across groups existed in both the Paris and TRIAD cohorts (*P* < 0.0001). No significant differences in sex between groups were observed for any of the three cohorts. In both the Paris and TRIAD cohorts, significant differences in *APOE*-ε4 allele frequency and MMSE score existed between groups. Full demographic information, clinical features and biomarker concentrations are shown in Table 1 (Paris and TRIAD cohorts) and Supplementary Table 1 (Discovery cohort).

### CSF p-tau205 and CSF p-tau202 across diagnostic groups

**Fig. 1:**
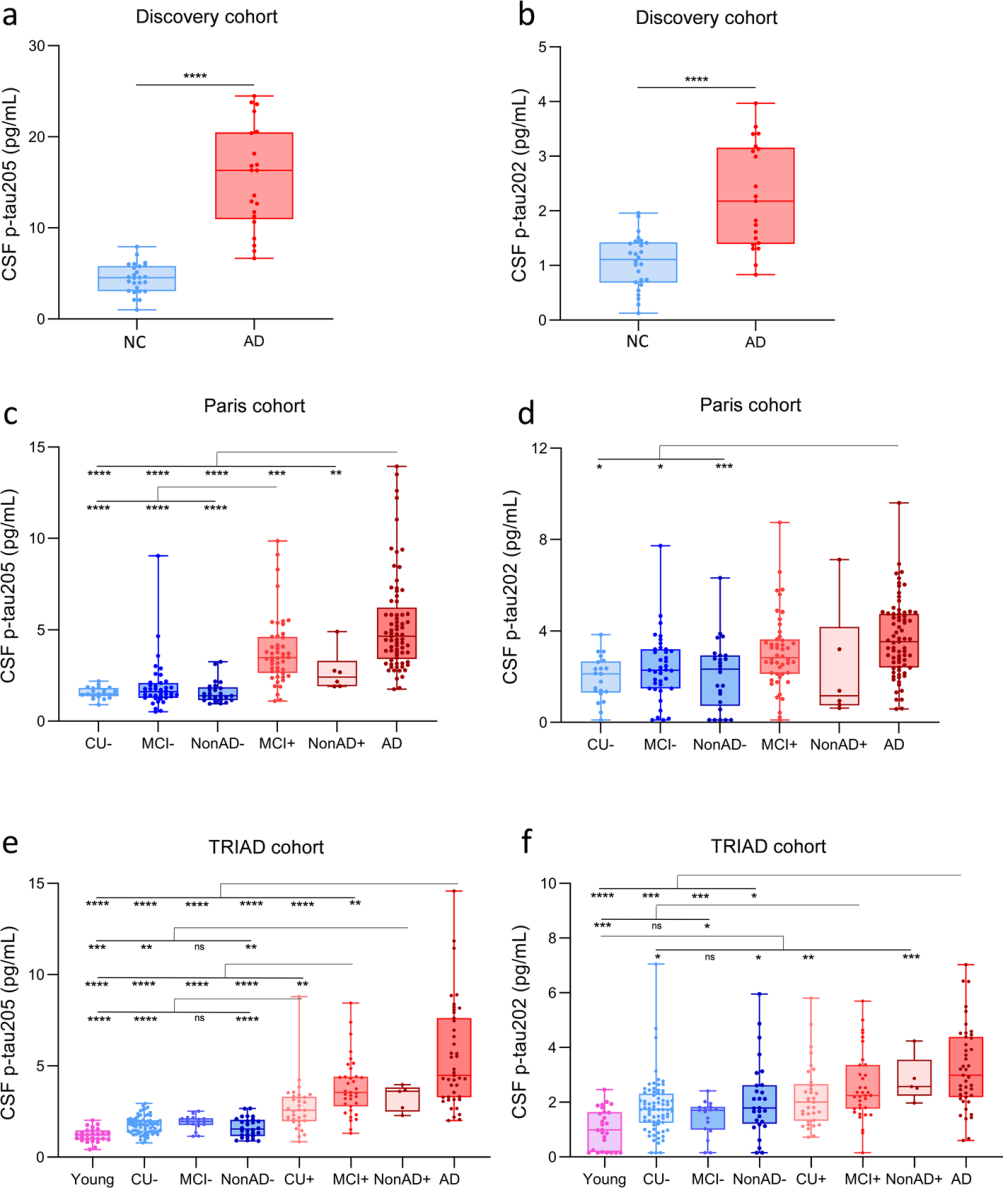
CSF p-tau205 and p-tau202 levels across diagnostic groups. In the Discovery cohort, **a** CSF p-tau205 and **b** CSF p-tau202 were increased in AD compared with control cases. In the Paris cohort, **c** CSF p-tau205 was increased in MCI+ and AD compared with CSF Aβ− groups, **d** whereas CSF p-tau202 was only significantly increased in AD compared with CSF Aβ− groups. In the TRIAD cohort, **e** CSF p-tau205 was increased across CSF Aβ+ groups compared with CSF Aβ− groups, **f** while high levels of CSF p-tau202 were mostly circumscribed to the AD group. *Data information*: Boxplots show the median, IQR and all participants. Participants are colour-coded based on the presence (red) or absence (blue) of CSF amyloidosis measured with Lumipulse CSF Aβ42/40. *P*-values were determined using Mann–Whitney *U* test and one-way ANOVA adjusted by age and sex, followed by Bonferroni-corrected post hoc comparison (*P < 0.05, **P < 0.01, ***P < 0.001, ****P < 0.0001)

In the Discovery cohort, CSF p-tau205 and p-tau202 were increased in AD compared with neurological controls (*P* < 0.0001) (Fig. 1a and b). In the Paris cohort, CSF p-tau205 was increased in AD participants when compared with all CSF Aβ- groups (CU-, MCI−, nonAD-; *P* < 0.0001 for all), with MCI+ (*P* < 0.001) and nonAD+ (*P* < 0.01). CSF p-tau205 concentration was also higher in MCI+ compared with all CSF Aβ- groups (CU−, MCI−, nonAD−; *P* < 0.0001 for all) (Fig. 1c). CSF p-tau202 was only increased in AD compared with CSF Aβ- groups (CU−, MCI−, nonAD−; *P* < 0.05 for all) (Fig. 1d). In the TRIAD cohort, CSF p-tau205 was increased in AD and MCI+ compared with CSF Aβ- groups (*P* < 0.0001 for all). Additionally, CSF p-tau205 levels were higher in CU+ and nonAD+ compared with CU- and nonAD- (*P* < 0.01 for both). Between CSF Aβ-positive groups, CSF p-tau205 was increased in AD compared with CU+ (*P* < 0.0001) and MCI+ (*P* < 0.01), and in MCI+ compared with CU+ (*P* < 0.01) (Fig. 1e). CSF p-tau202 was significantly higher in AD compared with all CSF Aβ− groups (*P* < 0.05, for all), and in MCI+ compared with young and MCI− cases (*P* < 0.05, for all). CSF p-tau202 levels in both CU+ and nonAD+ were only increased when compared with young subjects (*P* < 0.01, for all) (Fig. 1f).

In the discovery cohort, both CSF p-tau205 and p-tau202 showed high performance identifying AD cases (AUC_205_ = 99.5%, AUC_202_ = 85.9%), but CSF p-tau205 performed significantly better (DeLong´s test, *P* < 0.01) (Supplementary Fig. 2). In the Paris cohort, CSF p-tau205 showed high diagnostic accuracy discriminating AD, nonAD+ and MCI+ from CU-, MCI− and nonAD− (AD: AUCs = 94.2–99.3%, nonAD+ : AUCs = 81.6–96.0%, MCI+ : AUCs = 87.1–94.1%) (Supplementary Fig. 4a, 4c and 4e). In contrast, CSF p-tau202 showed modest accuracies in all three scenarios (AD: AUCs = 72.7–81.6%, nonAD+ : AUCs = 50.7–61.5%, MCI+ : AUCs = 62.8–72.8%) (Supplementary Fig. 4b, d and f). In all cases, the performance of CSF p-tau205 was higher than that of CSF p-tau202 (DeLong: *P* < 0.05 for all; except nonAD+ vs MCI−). In the TRIAD cohort, CSF p-tau205 displayed high accuracies discriminating AD, nonAD+ and MCI+ from CSF Aβ-groups (AD: AUCs = 97.4–99.9%, nonAD+ : AUCs = 96.2–100%, MCI+ : AUCs = 91.9–98.5%) (Supplementary Fig. 5a, c and e). When discriminating CU+ from CSF Aβ- groups, CSF p-tau205 showed moderate to high accuracies (CU+ : AUCs = 75.6–92.5%) (Supplementary Fig. 5g). CSF p-tau202 AUC values were for the most part lower than those of CSF p-tau205: (AD: AUCs = 73.3–92.3%, nonAD+ : AUCs = 72.9–98.5%, MCI+ : AUCs = 62.6–86.5%, CU+ : AUCs = 53.2–80.0%) (Supplementary Fig. 5b, d, f and h).

### CSF p-tau205 and CSF p-tau202 across AT groups

**Fig. 2:**
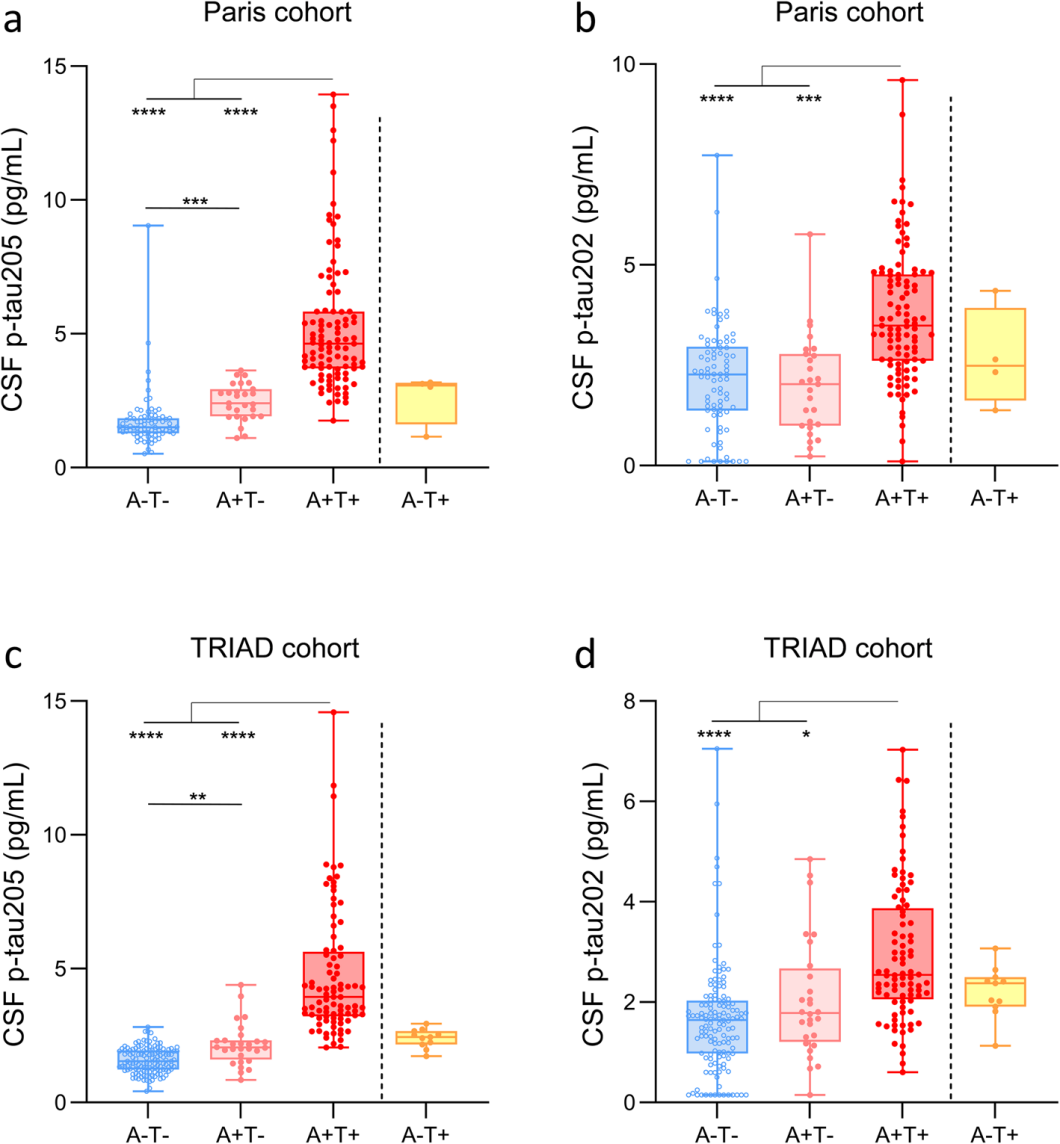
CSF p-tau205 and p-tau202 levels across CSF AT groups. In the Paris cohort, **a** CSF p-tau205 increased progressively across the groups stratified by Aβ (A) and tau (T) positivity, **b** whereas CSF p-tau202 was only increased in A+T+ group. In the TRIAD cohort, **c** CSF p-tau205 increased in a stepwise manner across AT groups **d** while high levels of CSF p-tau202 were only present in A+T+. *Data information*: Boxplots show the median, IQR and all participants. Participants colour-coded based on the presence (red) or absence (blue) of CSF amyloidosis measured with Lumipulse CSF Aβ42/40. *P*-values were determined using one-way ANOVA adjusted by age and sex, followed by Bonferroni-corrected post hoc comparison (*P < 0.05, **P < 0.01, ***P < 0.001, ****P < 0.0001)

In the Paris and TRIAD cohorts, CSF p-tau205 increased progressively across the AT groups stratified using CSF core biomarkers (Fig. 2a and c): a slight yet significant increase was observed from A−T− to A+T− (*P* < 0.01, for both), followed by a pronounced increase between A+T− and A+T+ (*P* < 0.0001, for both). Contrarily, CSF p-tau202 levels in Paris and TRIAD cohorts were only significantly increased in A+T+ cases compared to A−T− and A+T− (*P* < 0.05, for all) (Fig. 2b and d). In the TRIAD cohort, AT groups were also examined using Aβ and tau pathology indexed by PET for stratification. CSF p-tau205 increased gradually from A−T− to A+T−, and from A+T− to A+T+ (*P* < 0.0001, for all) (Supplementary Fig. 7a). CSF p-tau202 was only increased in A+T+ individuals (*P* < 0.05, for all) (Supplementary Fig. 7b). Both CSF p-tau205 and p-tau202 displayed the highest concentration in Aβ-PET negative and tau-PET positive cases (A−T+).

In both cohorts, CSF p-tau205 showed high performance discriminating CSF stratified A+T+ from A+T− (AUCs = 92.7–93.7%) and A−T− (AUCs = 97.4–99.3%), and moderate-to-high when discriminating A−T− from A+T− (AUCs = 74.7–82.7%) (Supplementary Fig. 6a and c). CSF p-tau202 performed better discriminating A+T+ from A+T− (AUCs = 70.8–81.6%) and A−T− (AUCs = 78.1–80.3%) than distinguishing A−T− from A+T− (AUCs = 54.9–59.9%) (Supplementary Fig. 6b and d). In both cohorts, the performance of CSF p-tau205 was superior to that of CSF p-tau202 when discriminating AT groups (*P* < 0.05, for all). In PET defined AT groups, CSF p-tau205 showed high performance discriminating AT groups, whereas CSF p-tau202 displayed comparatively more modest AUC values (Supplementary Fig. 7c and d). However, while CSF p-tau205 statistically outperformed CSF p-tau202 discriminating A−T− vs A+T− and A−T− vs A+T+ (*P* < 0.0001, for all), no significant differences were observed between both fluid markers when discriminating A+T− and A+T+.

### Associations of CSF p-tau205 and p-tau202 with Aβ pathology indexed by Aβ-PET

**Fig. 3:**
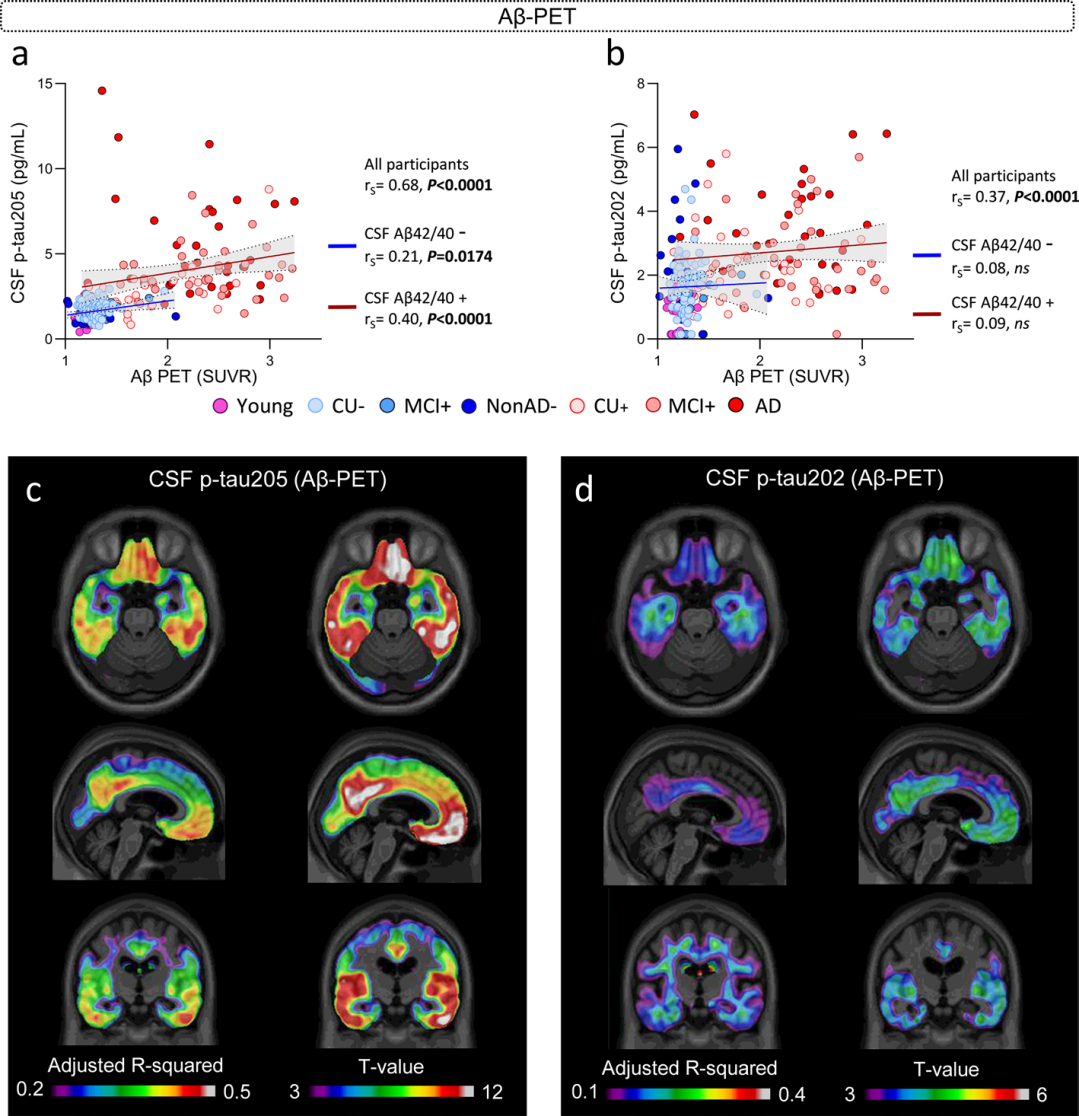
CSF p-tau205 and p-tau202 association with Aβ pathology measured by Aβ-PET (TRIAD cohort). **a** CSF p-tau205 correlated with global Aβ-PET SUVRs across all cases and CSF Aβ+ and Aβ− participants. **b** CSF p-tau202 only correlated with global Aβ-PET SUVRs across all participants. **c** CSF p-tau205 and **d** CSF p-tau202 displayed regional associations with Aβ-PET. *Data information*: Participants colour-coded based on the presence (red) or absence (blue) of CSF amyloidosis measured with Lumipulse CSF Aβ42/40. Spearman’s rank correlation is displayed for all participants, CSF Aβ+ and Aβ− groups. Simple linear regression with 95% confidence intervals of CSF Aβ+ and Aβ− groups is also presented. Voxel maps display the adjusted R-squared and t values of the linear associations between CSF biomarkers and [^18^F]AZD4694, adjusted by age and sex

In the subset of TRIAD samples with available Aβ-PET imaging (Supplementary Table 5), CSF p-tau205 showed a strong correlation with Aβ-PET SUVRs (r_S_ = 0.68, *P* < 0.0001) (Fig. 3a). The same was true for CSF p-tau202, but the strength of the correlation was weaker than that of CSF p-tau205 (r_S_ = 0.37, *P* < 0.0001) (Fig. 3b). When stratified by CSF Aβ42/40, CSF p-tau205 showed moderate correlation with Aβ-PET SUVRs in CSF Aβ+ cases (r_S_ = 0.40, *P* < 0.0001) and weak correlation in CSF Aβ- participants (r_S_ = 0.21, *P* < 0.05). CSF p-tau202 did not correlate with Aβ-PET SUVRs in participants stratified by CSF Aβ42/40.

When participants were stratified into Aβ-PET negative and positive groups, both CSF p-tau205 and p-tau202 were significantly increased in the latter group (*P* < 0.0001), but CSF p-tau205 displayed higher accuracy than CSF p-tau202 when discriminating the two groups (AUC_205_ = 90.3%, AUC_202_ = 71.1%; DeLong: *P* < 0.0001) (Supplementary Fig. 8).

In the voxel-wise analysis, the association between Aβ-PET and the two CSF biomarkers was localized in AD-related regions: the posterior cingulate, praecuneus, temporal and frontal cortices (Fig. 3c and d). However, results suggest a stronger association between CSF p-tau205 and Aβ-PET compared with CSF p-tau202, which is evidenced by the higher adjusted-*R*^2^ values and higher significant T-values that also encompassed wider regions for CSF p-tau205.

### CSF p-tau205 and CSF p-tau202 associations with tau pathology indexed by tau-PET

**Fig. 4:**
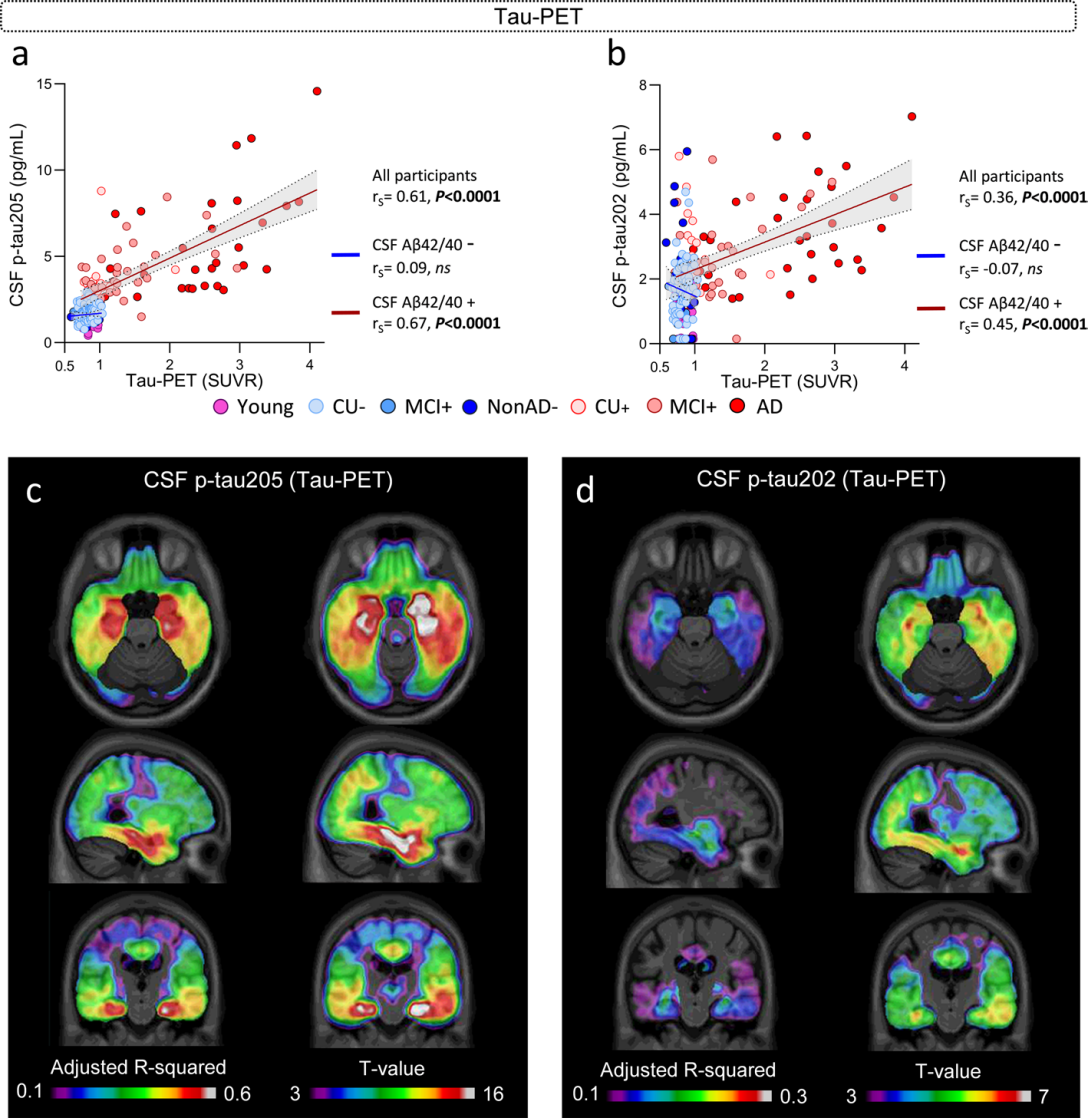
CSF p-tau205 and p-tau202 association with tau pathology measured by tau-PET (TRIAD cohort). **a** CSF p-tau205 and **b** CSF p-tau202 correlated with global tau-PET SUVRs across all cases and CSF Aβ+ participants. **c** CSF p-tau205 and **d** CSF p-tau202 displayed regional associations with tau-PET. *Data information*: Participants colour-coded based on the presence (red) or absence (blue) of CSF amyloidosis measured with Lumipulse CSF Aβ42/40. Spearman’s rank correlation is displayed for all participants, CSF Aβ+ and Aβ- groups. Simple linear regression with 95% confidence intervals of CSF Aβ+ and Aβ− groups is also presented. Voxel maps display the adjusted R-squared and t values of the linear associations between CSF biomarkers and [18F]MK6240, adjusted by age and sex

In the subset of TRIAD samples with available tau-PET imaging (Supplementary Table 5), CSF p-tau205 and p-tau202 significantly correlated with tau-PET SUVRs, but the strength of the correlation was stronger for CSF p-tau205 (r_S_ = 0.61, *P* < 0.0001) than for p-tau202 (r_S_ = 0.36, *P* < 0.0001) (Fig. 4a and b). CSF p-tau205 strongly and significantly associated with tau-PET SUVRs in CSF Aβ+ cases (r_S_ = 0.67, *P* < 0.0001), but not in CSF Aβ− participants (*P* > 0.05). The same was observed for CSF p-tau202, but the correlation with tau-PET in CSF Aβ+ patients was more moderate (r_S_ = 0.45, *P* < 0.0001). We further investigated the association of CSF p-tau205 and p-tau202 with tau-PET SUVRs across diagnostic groups. CSF p-tau205 displayed moderate-to-strong correlations with tau-PET SUVRs across diagnostic groups within the AD *continuum*, and these progressively increased in strength from CU+ (r_S_ = 0.43, *P* < 0.0147**)** to MCI+ (r_S_ = 0.56, *P* < 0.001), and from MCI+ to AD (r_S_ = 0.64, *P* < 0.0001) (Supplementary Fig. 9a). CSF p-tau202 correlated with tau-PET SUVRs only in the AD group, showing a moderate correlation (r_S_ = 0.41, *P* = 0.021) (Supplementary Fig. 9b).

**Fig. 5:**
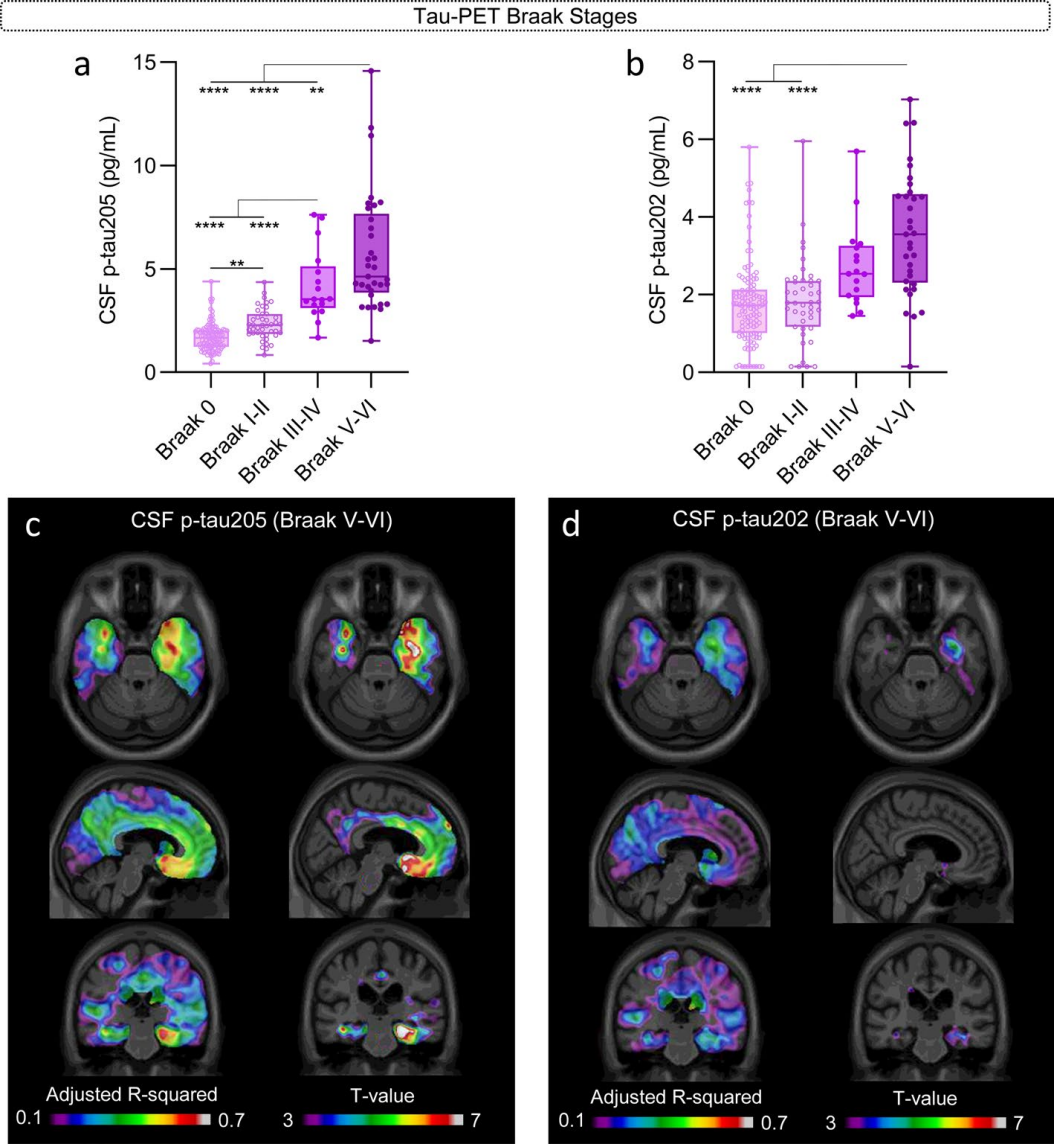
CSF p-tau205 and p-tau202 concentrations across tau-PET Braak stages and regional association with tau-PET Braak V-VI. **a** CSF p-tau205 increased progressively across tau-PET Braak stages, whereas **b** CSF p-tau202 was only increased in tau-PET Braak V-VI individuals. Regional association between **c** CSF p-tau205 and **d** CSF p-tau202 with tau-PET Braak V-VI participants. *Data information*: Boxplots show the median, IQR and all participants. *P*-values were determined using one-way ANOVA adjusted by age and sex, followed by Bonferroni-corrected post hoc comparison (*P < 0.05, **P < 0.01, ***P < 0.001, ****P < 0.0001). Voxel maps display the adjusted *R*-squared and *t* values of the linear associations between CSF biomarkers and [18F]MK6240 at Braak V-VI, adjusted by age and sex

Both CSF p-tau205 and p-tau202 were significantly increased in tau-PET positive individuals compared with tau-PET negative (*P* < 0.0001), but CSF p-tau205 showed higher accuracy than CSF p-tau202 discriminating the two groups (AUC_205_ = 94.5%, AUC_202_ = 81.4%; DeLong: *P* < 0.0001) (Supplementary Fig. 10), and both CSF p-tau biomarkers displayed higher discriminatory accuracies than for Aβ-PET described in the previous section. When stratifying participants into tau-PET Braak stages, CSF p-tau205 showed a stepwise increase across tau-PET Braak stages, displaying significant increases from Braak 0 to I-II (*P* < 0.01), from Braak I-II to III-IV (*P* < 0.0001), and from Braak III-IV to V-VI (*P* < 0.0001) (Fig. 5a). The increase in CSF p-tau205 concentration was especially pronounced at Braak III-IV and V-VI. CSF p-tau202 also displayed a stepwise increase across tau-PET Braak stages but was only significantly increased in Braak stage V-VI compared with Braak stages 0 and I-II (*P* < 0.0001) (Fig. 5b).

At the voxel level, the association between tau-PET with CSF p-tau205 and p-tau202 was more prominent in temporal regions, with highest adjusted-*R*^2^ values localized on the medial temporal regions (Fig. 4c and d). Once again, the model including CSF p-tau205 better explained tau-PET than the one with CSF p-tau202, given the higher adjusted-*R*^2^ values and the wider significant brain regions observed for CSF p-tau205. In addition, for CSF p-tau205, the adjusted-*R*^2^ values found with tau-PET were also larger as compared to Aβ-PET, which may suggest a closer association between CSF p-tau205 and tau-PET than with Aβ-PET. We also performed the same linear models within groups of participants at different Braak stages (within Braak I-II, Braak III-IV and Braak V-VI independently of each other). The association between CSF p-tau205 and p-tau202 with tau-PET was found to be significant only within the group of Braak V-VI stages, and only for p-tau205, suggesting a potential association between this biomarker and advanced tau pathology (Fig. 5c and d).

### CSF p-tau205 and p-tau202 explained by tau-PET

**Fig. 6:**
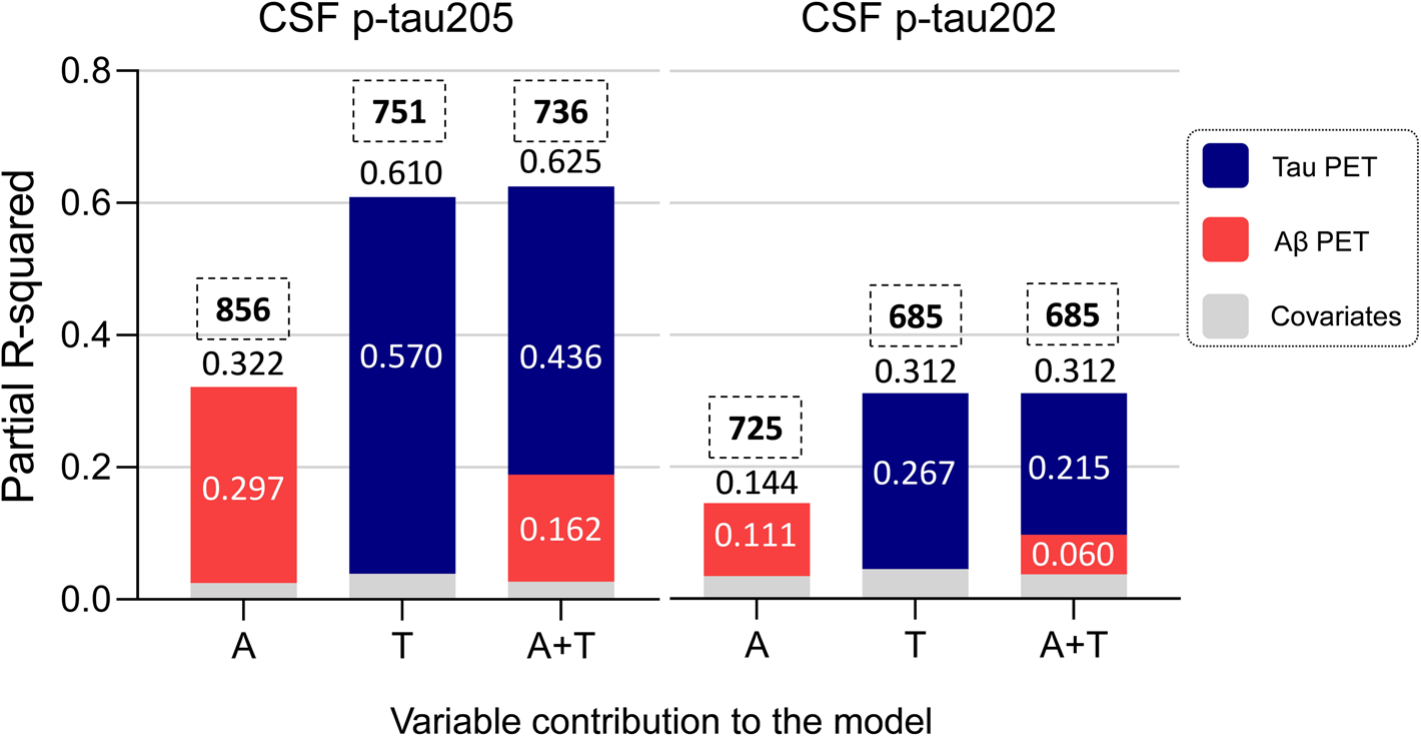
Proportion of variation in CSF p-tau205 and p-tau202 levels explained by Aβ and tau pathology measured by PET (TRIAD cohort). The performance of three regression models (Aβ-PET: A, tau-PET: T, Aβ and tau-PET: A+T) predicting CSF p-tau205 and p-tau202 concentrations was evaluated. The best model predicting the variation in the concentration of CSF p-tau205 was A+T, whereas for CSF p-tau202 it was T. *Data information:* Each bar plot represents one model. Independent variables included Aβ-PET (A, in red) and tau-PET (T in blue). All models include age and sex as covariates (represented in grey). AIC of each model is displayed on top of each bar plot, within a dashed square. R-squared values for each model are displayed on top of the respective bar plot, whereas the partial R-squared of each variable within the model is presented inside the bar plot

We investigated the proportion of variation in CSF p-tau205 and p-tau202 explained by Aβ-PET and tau-PET using regression models. First, we performed independent multivariable regression analyses to determine which variables optimally described the variation of CSF p-tau205 and p-tau202 concentrations across all groups. We generated three models using Aβ-PET and tau-PET as independent variables, and CSF p-tau205 or p-tau202 as dependent variables (using age and sex as covariates). The regression models included (i) Aβ-PET (A), (ii) tau-PET (T), (iii) Aβ-PET and tau-PET (A+T). The assessment of the models was determined based on the R-squared (*R*^2^) and the Akaike criterion (AICc, ΔAIC > 2 was considered significant). According to this, the model that better explained CSF p-tau205 concentrations was A+T, (*R*^2^ = 0.625, AIC = 736, ΔAIC = 15). We investigated the proportion of variation explained by each of the independent variables in the A+T model (partial *R*^2^): T accounted for a partial R^2^ = 0.436 (69.7%), followed by A with a partial *R*^2^ = 0.162 (25.9%) (Fig. 6). For CSF p-tau202 both the T and the A+T model showed the same *R*^2^ and AIC (*R*^2^ = 0.312, AIC = 685), therefore the simplest model, that is the T only model, was considered the best-fitting and most parsimonious model (Fig. 6). In subset of the TRIAD cohort (*n* = 202) where CSF p-tau181, p-tau217 and p-tau231 measurements were available (Supplementary Table 6), we compared their proportion of variation explained by Aβ-PET and tau-PET, and compared with CSF p-tau202 and p-tau205. For all biomarkers, the best-fitting model was A+T. Among all investigated CSF p-tau biomarkers, p-tau205 showed the highest *R*^2^ (A + T model: *R*^2^ = 0.636), with T accounting for the highest partial *R*^2^ = 0.32 (Supplementary Fig. 11).

As a complementary assessment, we performed mediation analysis to verify the direct and indirect contribution of PET biomarkers to the CSF biomarker levels. When considering tau-PET as a mediator in the association between Aβ-PET and CSF p-tau205 (adjusting for age and sex), we found Aβ-PET to have significant direct and indirect (through tau-PET) effects on CSF p-tau205 (*β*_direct_ = 0.32, *P*_direct_ < 0.001; *β*_indirect_ = 0.29, *P*_indirect_ < 0.001), whilst the direct effect of tau-PET alone was 0.49 (*P* < 0.001). For CSF p-tau202, however, the effect of Aβ-PET was totally mediated by tau-PET (*β*_direct_ = 0.07, *P*_direct_ = 0.22; *β*_indirect_ = 0.18, *P*_indirect_ < 0.001) and again tau-PET alone had a larger effect on this biomarker (*β* = 0.32, *P* < 0.001).

### CSF p-tau205 and p-tau202 association with neurodegeneration and cognition

A subset of 213 individuals in TRIAD cohort had available structural MRI measurements (Supplementary Table 7). Both CSF p-tau205 and p-tau202 showed significant and negative correlations with global measures of grey matter quantified with voxel-based morphometry (VBM) (CSF p-tau205: r_S_ = − 0.36, CSF p-tau202: r_S_ = − 0.33; *P* < 0.0001 for both) (Supplementary Fig. 12a and b). When stratified by CSF Aβ42/40, CSF p-tau205 showed a negative correlation with grey matter volume in both Aβ+ (r_S_ = − 0.29, *P* < 0.01) and Aβ- cases (r_S_ = − 0.25, *P* < 0.01). For CSF p-tau202, similar correlation was present only in Aβ- cases (r_S_ = − 0.27, *P* < 0.01). At the voxel level, significant associations were restricted to the posterior cingulate and medial temporal cortices and mostly detected for CSF p-tau205 (Supplementary Fig. 12c and d).

In both the Paris and TRIAD cohorts, cognitive assessments using MMSE were available in 207 and 218 participants, respectively. CSF p-tau205 showed a significant and negative correlation with MMSE in Paris (r_S_ = − 0.38; *P* < 0.0001) and TRIAD cohort (r_S_ = − 0.40; *P* < 0.0001). CSF p-tau202 also correlated with MMSE scores in the Paris (r_S_ = − 0.20; *P* < 0.01) and TRIAD (r_S_ = − 0.29; *P* < 0.0001) cohorts, but these were weaker than those of CSF p-tau205. When stratified by CSF Aβ42/40, CSF p-tau205, but not CSF p-tau202, correlated with MMSE in Aβ+ participants (Paris cohort: r_S_ = − 0.23, *P* < 0.05; TRIAD cohort: r_S_ = − 0.32, *P* < 0.01).

## Discussion

In this study, we report the development of the first two immunoassays measuring p-tau205 and p-tau202 concentrations in CSF and investigate their biomarker potential for the in vivo detection of tau pathology in AD. Our findings are concordant across the cohorts and indicate that (i) CSF p-tau205 increases progressively across the AD *continuum* (including preclinical AD stages), whereas CSF p-tau202 is only increased in AD (and A+T+) cases; (ii) CSF p-tau205 and p-tau202 are more tightly associated with tau pathology than Aβ pathology, both in terms of global PET measures and at the voxel level; (iii) tau pathology is the most prominent contributor to CSF p-tau205 and p-tau202 variance; (iv) CSF p-tau205 and p-tau202 correlate with grey matter atrophy globally and at the voxel level; and (v) CSF p-tau205 and p-tau202 are associated with lower cognitive performance. Overall, this study suggests that while both CSF p-tau205 and p-tau202 are specific markers of tau pathology in AD, CSF p-tau205 has an overall superior performance with less overlap between groups, and a stronger association with tau-PET.

P-tau species arguably represent the most promising fluid AD biomarkers, as they are highly specific for AD, they emerge early during asymptomatic AD stages and are tightly associated with both Aβ and tau accumulation. However, while p-tau is classified as a tau pathology or “T” biomarker according to the AT(N) framework [15], accumulating evidence suggests that p-tau is not merely reflective of neurofibrillary pathology in AD. Fluid p-tau species are tightly associated with cerebral amyloidosis assessed by CSF or PET biomarkers during preclinical AD, starting to increase when only subtle abnormalities in CSF Aβ42/40 are detectable [20, 45]. Moreover, recent studies indicate that p-tau species are more associated with Aβ-PET than tau-PET [25, 49], and in post-mortem confirmed samples, p-tau measurements are more strongly associated with Aβ plaques than tau tangles [42]. Hence, given the tight association between fluid p-tau and Aβ pathology across the AD *continuum*, it is difficult to establish whether increases in p-tau biomarkers (e.g., p-tau181, p-tau217 and p-tau231) are indicative of Aβ or tau deposition in the brain. Thus, there is a great need for fluid biomarkers capable of specifically reflecting and tracking aggregated tau pathology in brain. In this context, CSF p-tau205 may represent a useful biomarker alternative to current p-tau species for tracking tau pathology in AD brain.

In both the Paris and TRIAD cohorts, CSF p-tau205 concentrations were higher in all CSF Aβ+ groups compared with CSF Aβ− groups and showed a continuous increase along the AD *continuum* (and CSF defined AT groups)*,* in concordance with previous results from MS studies [5, 8, 13]. Interestingly, in the TRIAD cohort, CSF p-tau205 was significantly increased in preclinical AD. In a previous study using an antibody-free MS method for the quantification of CSF p-tau in TRIAD and BioFINDER-2 cohorts, higher albeit not significant concentrations of CSF p-tau205 were observed in CU+ compared with CU− [13]. Thus, our results further expand previous studies by demonstrating that CSF p-tau205 starts increasing during preclinical AD stages. However, because the sample size of CU groups was rather small in the TRIAD cohort, further studies investigating the emergence of CSF p-tau205 and its biomarker potential in preclinical AD are warranted. We also observed that CSF p-tau205 has high accuracies when discriminating diagnostic and AT groups (defined by either CSF or PET). Contrarily, high CSF p-tau202 levels were most frequently observed in AD and A+T+ cases, suggesting that this biomarker increases late on the AD *continuum*. This agrees with one previous publication which reported increased levels with advancing stages of AD [13], but contrasts with other reports that showed decreases with disease progression using the p-tau202/non-phospho ratio [6, 8]. A possible explanation for these discrepancies could be that in the late stages of the disease, when CSF p-tau202 is elevated, the levels of CSF non-phosphorylated tau rise as well, and if this increase occurs in a larger magnitude than that of CSF p-tau202, the normalization with the non-phosphorylated peptide might show a decrease. However, with a high degree of overlap across diagnostic and AT groups (which has been observed by us and others [13]), the performance of CSF p-tau202 was overall lower than that of CSF p-tau205 in both cohorts.

Across all TRIAD cohort participants, CSF p-tau205 displayed strong correlations with Aβ and tau accumulation measured with PET in regions typical for AD pathology, and these were stronger than those of CSF p-tau202. Additionally, the strength of the correlations between CSF p-tau205 and p-tau202 with Aβ and tau-PET across all participants were similar, in concordance with previous results [13]. However, when stratified according to CSF Aβ42/40 status, both biomarkers were more strongly associated with tau-PET than Aβ-PET. Moreover, across diagnostic groups CSF p-tau205 only correlated with tau-PET in CSF Aβ+ groups, and these were increasingly stronger with disease progression (CU+  < MCI+ < AD). CSF p-tau202 only significantly correlated with tau-PET in AD cases. These results indicate that CSF p-tau205 correlation with tau PET strengthens with advancing disease stages, while CSF p-tau202 is associated with tau-PET only in late AD. Furthermore, both p-tau biomarkers displayed higher accuracies discriminating positivity for tau-PET than Aβ-PET, suggesting the increase in CSF p-tau205 and p-tau202 is closer to tau-PET positivity threshold rather than Aβ-PET becoming abnormal. Moreover, across PET defined AT groups, both CSF biomarkers displayed the highest concentrations in A–T+ cases, suggesting a strong association with in vivo tau burden. Further supporting their close link with tau pathology, these two p-tau biomarkers were more strongly associated with tau than Aβ burden at the voxel level. This was particularly true for CSF p-tau205, which displayed substantially larger adjusted-*R*^2^ values with tau-PET. Moreover, voxel-wise analysis demonstrated that CSF p-tau205 (but not p-tau202) was significantly associated with tau-PET only in Braak V-VI participants. These results align with a previous publication reporting similar brain tissue concentrations of p-tau202/p-tau205 in healthy controls and AD in Braak stage I-IV, but increased p-tau202/p-tau205 in Braak V/VI [34]. This work also described that p-tau202/p-tau205 were focally present in hippocampus in Braak stage III-IV but disseminated to temporal regions at later stages (V-VI), where we also observed the most prominent associations with CSF p-tau202 and p-tau205 and tau-PET.

We further demonstrated the association of CSF p-tau205 and p-tau202 with tau deposition by determining the proportion of variance of both CSF biomarkers explained by Aβ-PET and tau-PET. The model that better explained the variation of CSF p-tau205 was the A+T model, and within this model, tau-PET contributed with a 70% of the variance. For CSF p-tau202, both the T and the A+T models showed the same *R*^2^, so the T only model was considered as the best fitting model for being the simplest (tau-PET contributed with 86% of variance). These results confirm that p-tau205 and p-tau202 levels are both reflecting tau brain pathology, but with subtle differences. Aβ-PET showed direct and indirect (through tau-PET) effects on CSF p-tau205 concentrations, but tau-PET alone displayed a larger effect. Contrarily, Aβ-PET effect on CSF p-tau202 was completely mediated by tau-PET, with tau-PET alone showing a stronger effect. Altogether, these results align with (i) CSF p-tau205 subtly emerging in CU+ cases but showing the bulk of the increase in cognitively impaired Aβ+ cases, especially in late cases (that is AD, A+T+ and Braak V-VI), and with (ii) CSF p-tau202 being exclusively increased in late cases. Furthermore, these findings corroborate and expand recent evidence showing that plasma p-tau205 concentrations were mainly explained by tau pathology assessed with tau-PET [32]. In this context, recent reports have shown that fluid p-tau measurements such as p-tau181, p-tau217 and p-tau231 are similarly or more closely associated with Aβ pathology and tau pathology, determined using imaging biomarkers [25, 29, 30, 49] and in post-mortem confirmed samples [33, 42]. The availability of a small subset of TRIAD cohort participants with available CSF p-tau181, p-tau217 and p-tau231 measurements allowed the comparison of these p-tau species with CSF p-tau202 and p-tau205, resulting in p-tau205 showing not only the highest *R*^2^ of all tested models, but also displaying the highest partial *R*^2^ for tau pathology indexed by tau PET. Thus, previous and present results suggest that CSF p-tau205 might reflect better tau pathology across the AD *continuum* than other available p-tau biomarkers.

Finally, both CSF p-tau205 and p-tau202 associated with grey matter atrophy and correlated with cognitive function (MMSE). For CSF p-tau205, this agrees with previous publications using MS methods showing that p-tau205 is more strongly associated with grey matter volume than other phosphorylations on tau protein [5]. In addition, two recent papers reported that CSF p-tau205 had the strongest correlation with regional brain volumes compared to other p-tau species [7] and was the only phosphorylation associated with white matter loss [44]. This link with brain atrophy might also translate into a better association with cognition. Interestingly, post-mortem Braak stages have been shown to better correlate with disease progression and cognitive decline than Aβ Thal staging. The fact that CSF p-tau205 reflects tau pathology suggests it might be a better indicator of cognitive deterioration. In a previous work, CSF p-tau205 concentration was shown to have the strongest correlation among different phosphorylations with dementia severity assessed by Clinical Dementia Taking Sum of Boxes (CDR-SB) [7]. In the present study, we show that CSF p-tau205 correlated with MMSE scores in both Paris and TRIAD cohorts. In the case of CSF p-tau202, previous MS literature has shown weak correlations between brain volume loss and cognition with the CSF p-tau202 ratio, as observed here with the immunoassay measures [8].

This study is not exempt of limitations. First, variability among different tau-PET ligands has been described [51], and therefore, it would be of high interest to investigate whether our results with MK-6240 concord with different tracers and to which degree. Second, while tau-PET provides with a in vivo visualization of NFT pathology, this is not completely interchangeable with post-mortem Braak staging. Therefore, further studies using neuropathology-confirmed samples will be needed to fully stablish the relationship between CSF p-tau205 and p-tau202 and neurofibrillary pathology in AD. Third, we observed that CSF p-tau205 was subtly increased in CU+ participants. However, the sample size of this group was rather small and therefore further studies investigating the emergence of CSF p-tau205 in preclinical AD cases are needed. Finally, longitudinal studies would provide a better characterization of CSF p-tau205 and p-tau202 association with tau pathology and stage, neurodegeneration, and cognitive decline.

Recent advances in the development of anti-Aβ therapies for the treatment of AD strengthen the importance of fluid biomarkers not only for diagnostic purposes, but also as tools for screening/enriching participants in clinical trials and for monitoring drug effects. Recently, the TRAILBLAZER-2 donanemab trial succeeded in recruiting participants with intermediate tau-PET burden [39]. However, tau-PET presents significant cost, and requires highly specialized centres and personnel with expertise in this technique. Thus, a fluid biomarker reflecting tau burden and deposition would be highly valuable in clinical trials, as a cost-effective tool for screening and recruitment, participant stratification and staging, and for evaluating if disease progression has been tackled. Moreover, such a biomarker would represent a clinically relevant tool in clinical settings for AD diagnosis and patient management and monitoring. Our results indicate that CSF p-tau205 is a highly specific biomarker of AD, exhibiting a continuous and steep increase along with AD progression. Most importantly, it accurately reflects in vivo tau burden, thereby suggesting its potential usefulness identifying and staging tau pathology in AD. Thus, a high throughput method such as the digital immunoassays presented herein would be of great use in clinical settings and clinical trials. Regarding CSF p-tau202, the significant overlaps between groups, and comparatively weak association with AD pathological hallmarks restricts its potential as a useful biomarker in AD.

## Supplementary Information

Below is the link to the electronic supplementary material.

Supplementary file1 (DOCX 68504 kb)
