# Correction: CSF p-tau205: a biomarker of tau pathology in Alzheimer’s disease

**DOI:** 10.1007/s00401-025-02877-4

**Published:** 2025-04-21

**Authors:** Juan Lantero-Rodriguez, Laia Montoliu-Gaya, Andrea L. Benedet, Agathe Vrillon, Julien Dumurgier, Emmanuel Cognat, Wagner S. Brum, Nesrine Rahmouni, Jenna Stevenson, Stijn Servaes, Joseph Therriault, Bruno Becker, Gunnar Brinkmalm, Anniina Snellman, Hanna Huber, Hlin Kvartsberg, Nicholas J. Ashton, Henrik Zetterberg, Claire Paquet, Pedro Rosa-Neto, Kaj Blennow

**Affiliations:** 1 Department of Psychiatry and Neurochemistry, Institute of Neuroscience and Physiology, The Sahlgrenska Academy at the University of Gothenburg, Mölndal, Sweden; 2 Cognitive Neurology Center, Université de Paris Cité, GHU Nord APHP Hospital Lariboisière Fernand Widal, Paris, France; 3 Graduate Program in Biological Sciences: Biochemistry, Universidade Federal Do Rio Grande Do Sul (UFRGS), Porto Alegre, Brazil; 4 Montreal Neurological Institute, Montreal, QC Canada; 5 Department of Neurology and Neurosurgery, McGill University, Montreal, QC Canada; 6 Turku PET Centre, University of Turku, Turku University Hospital, Turku, Finland; 7 Clinical Neurochemistry Laboratory, Sahlgrenska University Hospital, Mölndal, Sweden; 8 Wallenberg Centre for Molecular and Translational Medicine, University of Gothenburg, Gothenburg, Sweden; 9 Department of Old Age Psychiatry, Maurice Wohl Clinical Neuroscience Institute, King’s College London, London, UK; 10 NIHR Biomedical Research Centre for Mental Health and Biomedical Research Unit for Dementia at South London and Maudsley NHS Foundation, London, UK; 11 Department of Neurodegenerative Disease, Queen Square Institute of Neurology, University College London, London, UK; 12 UK Dementia Research Institute, University College London, London, UK; 13 Hong Kong Center for Neurodegenerative Diseases, Hong Kong, China; 14 Wisconsin Alzheimer’s Disease Research Center, School of Medicine and Public Health, University of Wisconsin, University of Wisconsin-Madison, Madison, WI USA

**Correction: Acta Neuropathologica (2024) 147:12** 10.1007/s00401-023-02659-w

In this article, the following sentence with the term serine 205 was incorrectly published in the section Materials and methods under the subsection Simoa CSF p‑tau202 and p‑tau205 measurements:

Similarly, in the CSF p-tau205 immunoassay, a rabbit polyclonal antibody selective against phosphorylated tau at serine 205 (immunogen: synthesized human tau peptide around the phosphorylation site of serine205, ThermoFisher Scientific) was used as capture antibody with biotinylated Tau12 used for detection.

The correct term should be threonine 205, and the correct sentence should be as given below:

Similarly, in the CSF p-tau205 immunoassay, a rabbit polyclonal antibody selective against phosphorylated tau at threonine 205 (immunogen: synthesized human tau peptide around the phosphorylation site of threonine205, ThermoFisher Scientific) was used as capture antibody with biotinylated Tau12 used for detection.

Now, the original has been corrected.

