# Supplementary material for: CSF p-tau205: a biomarker of tau pathology in Alzheimer’s disease
Source: Acta Neuropathol. 2024 Jan 6;147(1):12. doi: 10.1007/s00401-023-02659-w (PMC10771353; doi:10.1007/s00401-023-02659-w)
Supplement: Supplementary file 1 — Supplementary file1 (DOCX 68504 kb) [file 401_2023_2659_MOESM1_ESM.docx]

**P-TAU205: A BIOMARKER OF TAU PATHOLOGY IN ALZHEIMER´S DISEASE**

Juan Lantero-Rodriguez^1*^, Laia Montoliu-Gaya^1*^, Andrea L. Benedet^1^, Agathe Vrillon^2^, Julien Dumurgier^2^, Emmanuel Cognat ^2^, Wagner S. Brum^1,3^, Nesrine Rahmouni^4,5^, Jenna Stevenson^4,5^, Stijn Servaes^4,5^, Joseph Therriault^4,5^, Bruno Becker^1^, Gunnar Brinkmalm^1^, Anniina Snellman^1,6^, Hanna Huber^1^, Hlin Kvartsberg^1,7^, Nicholas J. Ashton^1 ,8,9,10^, Henrik Zetterberg^1,7, 11,12,13,14^, Claire Paquet^2^, Pedro Rosa-Neto^4,5^and Kaj Blennow^1,7^.

Affiliations:

^1^ Department of Psychiatry and Neurochemistry, Institute of Neuroscience & Physiology, The Sahlgrenska Academy at the University of Gothenburg, Mölndal, Sweden

^2^ Université de Paris Cité, Cognitive Neurology Center, GHU Nord APHP Hospital Lariboisière Fernand Widal, Paris, France.

^3^ Graduate Program in Biological Sciences: Biochemistry, Universidade Federal do Rio Grande do Sul (UFRGS), Porto Alegre, Brazil

^4^ Montreal Neurological Institute, Montreal, QC, Canada

^5^ Department of Neurology and Neurosurgery, McGill University, Montreal, QC, Canada.

^6^ Turku PET Centre, University of Turku, Turku University Hospital, Turku, Finland.

^7^ Clinical Neurochemistry Laboratory, Sahlgrenska University Hospital, Mölndal, Sweden

^8^ Wallenberg Centre for Molecular and Translational Medicine, University of Gothenburg, Gothenburg, Sweden

^9^ Department of Old Age Psychiatry, Maurice Wohl Clinical Neuroscience Institute, King’s College London, London, UK

^10^ NIHR Biomedical Research Centre for Mental Health & Biomedical Research Unit for Dementia at South London & Maudsley NHS Foundation, London, UK

^11^ Department of Neurodegenerative Disease, Queen Square Institute of Neurology, University College London, London, UK

^12^ UK Dementia Research Institute, University College London, London, UK

^13^ Hong Kong Center for Neurodegenerative Diseases, Hong Kong, China

^14^ Wisconsin Alzheimer’s Disease Research Center, University of Wisconsin School of Medicine and Public Health, University of Wisconsin-Madison, Madison, WI, USA

*Contributed equally as first authors

**TABLE OF CONTENTS**

**1. SUPPLEMENTARY METHODS**

**CSF p-tau205 and CSF p-tau202:** Immunoassay development and validation.

**2. SUPPLEMENTARY TABLES**

**Supplementary Table 1.** Demographics of the 47 participants of the Discovery cohort.

**Supplementary Table 2**. Demographics of the 212 participants of the Paris cohort stratified using Lumipulse CSF Aβ42/40 and p-tau181 into AT groups.

**Supplementary Table 3.** Demographics of the 262 participants of the TRIAD cohort stratified using Lumipulse CSF Aβ42/40 and p-tau181 into AT groups.

**Supplementary Table 4.** Demographics of the 220 participants of the TRIAD cohort stratified using Aβ-PET and tau-PET into AT groups.

**Supplementary Table 5.** Demographics of the subset of TRIAD participants including Aβ-PET and tau-PET.

**Supplementary Table 6.** Demographics of the subset of TRIAD participants including CSF p-tau181, p-tau217 and p-tau231 measurements.

**Supplementary Table 7.** Demographics of the subset of TRIAD participants including neurodegeneration measurements using MRI.

**3. SUPPLEMENTARY FIGURES**

**Supplementary Figure 1.** Mass spectrometry validation of anti p-tau202 and p-tau205 antibodies.

**Supplementary Figure 2.** CSF p-tau205 and p-tau202 diagnostic performance in the Discovery cohort.

**Supplementary Figure 3.** Spearman’s rank correlation between CSF p-tau205 and p-tau202 assays with antibody-free mass spectrometry in the TRIAD cohort.

**Supplementary Figure 4.** CSF p-tau205 and p-tau202 diagnostic performance discriminating diagnostic groups in the Paris cohort.

**Supplementary Figure 5.** CSF p-tau205 and p-tau202 diagnostic performance discriminating diagnostic groups in the TRIAD cohort.

**Supplementary Figure 6**. CSF p-tau205 and p-tau202 diagnostic performance discriminating AT groups in the Paris and TRIAD cohorts.

**Supplementary Figure 7.** CSF p-tau205 and p-tau202 levels and performance across AT groups determined using Aβ and tau PET.

**Supplementary Figure 8.** CSF p-tau205 and p-tau202 concentrations and diagnostic performance in Aβ-PET negative and positive participants in the TRIAD cohorts.

**Supplementary Figure 9.** Spearman’s rank correlation between CSF p-tau205 and p-tau202 concentrations with tau-PET SUVRs across diagnostic groups in the TRIAD cohort.

**Supplementary Figure 10.** CSF p-tau205 and p-tau202 concentrations and diagnostic performance in in tau-PET negative and positive participants in the TRIAD cohort.

**Supplementary Figure 11.** Proportion of variation in CSF p-tau biomarkers explained by Aβ and tau pathology measured by PET (TRIAD cohort).

**Supplementary Figure 12.** CSF p-tau205 and p-tau202 associations with neurodegeneration.

**1. SUPPLEMENTARY METHODS**

**CSF p-tau205 and CSF p-tau202:** **immunoassay development and validation**

We developed two novel immunoassays (one for each phosphorylated residue) following the same N-terminal directed strategy successfully implemented in the development of previously reported p-tau181, p-tau217, p-tau231 and p-tau235 immunoassays [1, 5-7]. This design consists of partnering antibodies targeting non-phosphorylated tau epitopes located at the N-terminus and p-tau specific antibodies directed against mid-region phosphorylated residues. Capture antibodies (anti-p-tau202 and anti-p-tau205) were conjugated to magnetic homebrew carboxylated beads (Quanterix), and the detector antibody (N-terminal tau) was biotinylated in-house with EZ-Link^TM^ NHS-PEG4-Biotin (Thermo Scientific, USA). The optimized method was based on a 2-step protocol, where the beads conjugated to the capture antibody, the sample, and the detector antibody are first co-incubated, followed by a washing step. Subsequently, streptavidin-β-galactosidase and later resofurin β-D-galactopyranoside are added, and the resulting fluorescent signal is then measured and quantified.

The specificities of the two capture antibodies, i.e., anti-p-tau202 and anti-p-tau205, were assessed using immunoprecipitation (IP) followed by liquid chromatography coupled to mass spectrometry (LC-MS). To evaluate their specificity, the IP exposed the two anti-p-tau antibodies to a battery of tau peptides, specifically: two different peptides phosphorylated at position 202 ([tau 195-209-p202] and [tau 190-214-p202]), two different peptides phosphorylated at position 205 ([tau 195-209-p205] and [tau 190-214-p205]), and non-phosphorylated tau peptide ([tau195-209]). In brief, each individual IP was performed by coating 50 μL of Dynabeads M-280 sheep anti-rabbit IgG (Thermo Fisher Scientific, USA) with 4 μg of each anti-p-tau antibodies, following the manufacturer´s recommendations. Thus, 50 μL of antibody-coated beads were used to IP each sample, each of them containing 10 pmol of each peptide, diluted in phosphate-buffered saline or PBS (0.01 M phosphate buffer, 0.14 M NaCl; at pH 7.4) and including Triton X-100 to a final concentration of 0.05%. Samples were subsequently incubated overnight at 4 °C in a roller shaker. Samples were then washed and eluted using an automated magnetic particle processor (KingFisher, Thermo Fisher Scientific). Elution was accomplished using 100 μL of 0.5% formic acid. Samples were then dried in a vacuum centrifuge at RT and reconstituted in 50 mM ammonium bicarbonate containing trypsin (Promega) at a concentration of 3.33 µg/mL. Digestion was performed at 37 °C for 16 hours and then stopped by adding 2 µL 10% trifluoroacetic acid (TFA). Finally, eluates were again dried in a vacuum centrifuge at RT and later stored at -80 °C pending LC-MS analysis.

Nanoflow LC-MS was performed with a Dionex 3000 system coupled to a Q Exactive high resolution hybrid quadrupole–orbitrap mass spectrometer equipped with an electrospray ionization source (both Thermo Fisher Scientific, Inc.) as previously described with minor alterations [2, 3]. Briefly, samples immunoprecipitated were reconstituted in 7 μL 0.05% TFA/2% acetonitrile in deionized water and 6 μL was loaded onto an Acclaim PepMap C18 trap column (length 20 mm, internal diameter 75 μm, particle size 3 μm, pore size 100 Å, Thermo Fisher Scientific, Inc.) for desalting and sample clean-up. Sample loading buffer was 0.05% TFA in water. Separation was then carried out at a flow rate of 300 nL/min by applying a 50 min long linear gradient from 3% to 40% B using a reversed-phase Acclaim PepMap C18 analytical columns (length 150 mm, internal diameter 75 μm, particle size 2 μm, pore size 100 Å, Thermo Fisher Scientific, Inc.) where buffer A was 0.1% formic acid in water and buffer B was 0.1% formic acid/84% acetonitrile in water. The mass spectrometer was set to operate in data dependent mode using higher-energy collision-induced dissociation for ion fragmentation. The settings for both MS and MS/MS acquisition were: resolution setting 70 000, 1 microscan, target values 106, trap injection time 250 ms. LC-MS/MS acquisitions were processed using Mascot Daemon v2.6/Mascot Distiller v2.6.3 (both Matrix Science) for charge and isotope deconvolution before submitting searches using Mascot search engine v2.6.1. Searches were made against a custom made tau database; for processing and search settings, refer to the following previous publications [2, 3]. Quantitative analysis was performed using Skyline v22.2.0.257 (MaCoss Lab) [8]. Results of the IP-MS are presented in Supplementary Figure 1. The anti-ptau202 antibody displayed high intensity peaks exclusively when exposed to peptides phosphorylated at position 202 ([tau 195-209 p202] and [tau 190-214 p202]). It did not exhibit any reaction towards peptides phosphorylated at position 205 ([tau 195-209 p205] and [tau 190-214 p205]) or the non-phosphorylated peptide ([tau 195-209]). The anti-ptau205 antibody exhibited high intensity peaks only when exposed to peptides phosphorylated at position 205 ([tau 195-209 p205] and [tau 190-214 p205]) but did not react against peptides phosphorylated at position 202 ([tau 195-209 p202] and [tau 190-214 p202]) or the non-phosphorylated peptide ([tau 195-209]). This demonstrates the specificity of each antibody against its intended phosphorylated tau target, as well as the lack of cross-reactivity towards the other phosphorylations or the absence of them in the epitope region. In the discovery cohort, CSF p-tau205 and p-tau202 were significantly increased in AD and both showed high accuracies discriminating AD from neurological control cases (Figure 1a and 1b, Supplementary Figure 2).

We also investigated whether our Simoa methods for quantifying p-tau205 and p-tau202 in CSF correlated with previous measurements using an antibody-free MS method in the TRIAD cohort [4] (Supplementary Figure 3a and 3b). Quantification using these immunoassays correlated with the MS measurements of tryptic monophosphorylated p-tau202 and p-tau205 peptides. CSF p-tau205 showed strong and significant correlation with the MS measurements (r_S_=0.81, *P*<0.0001), whereas CSF p-tau202 displayed a moderate yet significant correlation with levels measured by MS (r_S_=0.40, *P*<0.0001) (Supplementary Figure 3a and 3b). It should be noted that while our two in-house developed immunoassays measure intact phosphorylated tau fragments elongating from the N-terminus to tau mid-region (thus requiring the presence of both the N-terminal and the phosphorylated epitopes), the MS method includes tryptic digestion of tau fragments in CSF, to later target and quantify monophosphorylated p-tau202 and p-tau205 peptides. Thus, while quantification of p-tau205 and p-tau202 residues in CSF using immunoassays and mass spectrometry is different, both types of measurements relevant and complementary: biologically meaningful differences between fragments can be identified with immunoassays as they respect their endogenous sequence, but mass spectrometry allows the measurement of a broader range of fragments (doesn’t have the two-epitope limitation of immunoassays) and allows simultaneous quantification of multiple p-tau species of interest.

**1. SUPPLEMENTARY TABLES**

**Supplementary Table 1. Demographics of the 47 participants of the Discovery cohort.**

| **Discovery cohort (n=47)** | **NC**  **(n=26)** | **AD**  **(n=21)** | ***P-*value** |
| --- | --- | --- | --- |
| **Age, years** | 57.7 (18.8) | 60.1 (16.1) | ns |
| **Males (%)** | 12 (46.2) | 7 (33.3) | ns |
| **INNOTEST CSF (pg/mL)** |  |  |  |
| **Aβ42** | 850.58 (283.17) | 436.43 (153.87) | ˂0.001 |
| **p-tau181** | 31.45 (11.77) | 121.81 (48.26) | ˂0.001 |
| **t-tau** | 268.23 (76.07) | 871.24 (382.55) | ˂0.001 |
| **GU CSF biomarkers (pg/mL)** |  |  |  |
| **p-tau202** | 1.06 (0.48) | 2.26 (0.96) | ˂0.001 |
| **p-tau205** | 4.45 (1.62) | 15.43 (5.69) | ˂0.001 |

Data is shown as mean (SD) or n (%), as appropriate. Kruskal Wallis test was used to compare age between groups and Pearson’s chi-square to compare sex frequencies between groups. Biomarkers levels were compared with using a Mann-Whitney *U* test. *Abbreviations:* Aβ42, β-amyloid 42; AD, Alzheimer’s disease; CSF, cerebrospinal fluid; GU, Gothenburg University Simoa assay; NC, neurological control; ns, non-significant; p-tau181, tau phosphorylated at threonine 181; p-tau202, tau phosphorylated at serine 202; p-tau205, tau phosphorylated at threonine 205; t-tau, total tau.

**Supplementary Table 2. Demographics of the 212 participants of the Paris cohort stratified using Lumipulse CSF Aβ42/40 and p-tau181 into AT groups.**

| **AT CSF (Paris cohort, n=212)** | **A-T-**  **(n=82)** | **A+T-**  **(n=27)** | **A+T+**  **(n=99)** | **A-T+**  **(n=4)** | ***P-*value** |
| --- | --- | --- | --- | --- | --- |
| **Age, years** | 66.16(9.19) | 71.07(7.20) | 71.88(8.41) | 64.00(10.49) | ˂0.001 |
| **Males (%)** | 35(42.7) | 9(33.3) | 37(37.4) | 2(50.0) | ns |
| ***APOE-*ε4 carriers (%)** | 15/81(18.5) | 14/27(51.9) | 65/98(66.3) | 0/4(0.0) | ˂0.001 |
| **MMSE score (available cases)** | 80 | 26 | 98 | 3 |  |
| **MMSE score** | 24.83(4.15) | 22.08(5.28) | 20.35(5.74) | 28.33(0.58) | ˂0.001 |
| **Lumipulse CSF (pg/mL)** |  |  |  |  |  |
| **Aβ42/40** | 0.09(0.01) | 0.05(0.01) | 0.04(0.01) | 0.09(0.01) | ˂0.001 |
| **p-tau181** | 33.63(10.60) | 48.06(10.00) | 116.28(55.69) | 65.98(26.81) | ˂0.001 |
| **t-tau** | 296.09(215.84) | 335.30(103.18) | 740.47(358.10) | 597.50(181.90) | ˂0.001 |
| **GU CSF biomarkers (pg/mL)** |  |  |  |  |  |
| **p-tau202** | 2.18(1.37) | 2.00(1.23) | 3.76(1.66) | 2.68(1.24) | ˂0.001 |
| **p-tau205** | 1.72(1.02) | 2.46(0.68) | 5.25(2.47) | 2.62(0.98) | ˂0.001 |

Data is shown as mean (SD) or n (%), as appropriate. Kruskal Wallis test was used to compare age between groups and Pearson’s chi-square to compare sex and *APOE*-ε4 frequencies between groups. MMSE and biomarkers levels were compared with a one-way ANOVA adjusted by age and sex. *Abbreviations*: Aβ42/40, ratio β-amyloid 42 and 40; A-T-, amyloid and tau negative; A+T-, amyloid positive tau negative; A+T+, amyloid and tau positive; A-T+, amyloid negative and tau positive; CSF, cerebrospinal fluid; GU, Gothenburg University Simoa assay; MMSE, Mini-Mental State Examination; ns, non-significant; p-tau181, tau phosphorylated at threonine 181; p-tau202, tau phosphorylated at serine 202; p-tau205, tau phosphorylated at threonine 205; t-tau, total tau.

**Supplementary Table 3. Demographics of the 262 participants of the TRIAD cohort stratified using Lumipulse CSF Aβ42/40 and p-tau181 into AT groups.**

| **AT CSF (TRIAD cohort, n=262)** | **A-T-**  **(n=135)** | **A+T-**  **(n=28)** | **A+T+**  **(n=88)** | **A-T+**  **(n=11)** | ***P-*value** |
| --- | --- | --- | --- | --- | --- |
| **Age, years** | 58.47(19.93) | 69.52(6.35) | 68.50(7.89) | 65.74(16.69) | ˂0.001 |
| **Males (%)** | 56(41.5) | 14(50.0) | 37(42.0) | 5(45.5) | ns |
| **Level of education, years** | 14.99(4.53) | 14.89(4.99) | 13.08(5.51) | 12.73(7.44) | ˂0.05 |
| ***APOE-*ε4 carriers (%)** | 24/128(18.8) | 15/26(57.7) | 42/78(53.8) | 4/9(44.4) | ˂0.001 |
| **MMSE score(available cases)** | 116 | 25 | 68 | 8 |  |
| **MMSE score** | 28.84(2.53) | 28.04(3.39) | 25.22(5.57) | 28.88(1.13) | ˂0.001 |
| **Lumipulse CSF (pg/mL)** |  |  |  |  |  |
| **Aβ42/40** | 0.09(0.01) | 0.06(0.01) | 0.04(0.01) | 0.09(0.01) | ˂0.001 |
| **p-tau181** | 29.81(9.25) | 39.23(9.69) | 106.60(63.40) | 58.92(5.33) | ˂0.001 |
| **t-tau** | 265.24(107.10) | 316.89(114.60) | 702.99(375.69) | 470.91(76.25) | ˂0.001 |
| **Aβ-PET (available cases)** | 119 | 25 | 74 | 9 |  |
| **SUVR** | 1.27(0.14) | 1.74(0.47) | 2.29(0.52) | 1.35(0.19) | ˂0.001 |
| **Tau-PET (available cases)** | 117 | 23 | 74 | 9 |  |
| **SUVR** | 0.83(0.09) | 0.96(0.33) | 1.73(0.89) | 0.83(0.06) | ˂0.001 |
| **VBM (available cases)** | 114 | 21 | 70 | 8 |  |
| **mm^3^** | 0.47(0.08) | 0.44(0.05) | 0.42(0.07) | 0.47(0.04) | ˂0.01 |
| **GU CSF biomarkers (pg/mL)** |  |  |  |  |  |
| **p-tau202** | 1.64(1.10) | 2.05(1.19) | 2.95(1.37) | 2.21(0.51) | ˂0.001 |
| **p-tau205** | 1.58(0.47) | 2.16(0.79) | 4.74(2.37) | 2.40(0.35) | ˂0.001 |

Data is shown as mean (SD) or n (%), as appropriate. Kruskal Wallis test was used to compare age between groups and Pearson’s chi-square to compare sex and *APOE*-ε4 frequencies between groups. Years of education, MMSE and biomarkers levels were compared with a one-way ANOVA adjusted by age and sex. *Abbreviations*: Aβ42/40, ratio β-amyloid 42 and 40; A-T-, amyloid and tau negative; A+T-, amyloid positive tau negative; A+T+, amyloid and tau positive; A-T+, amyloid negative and tau positive; CSF, cerebrospinal fluid; GU, Gothenburg University Simoa assay; MMSE, Mini-Mental State Examination; ns, non-significant; p-tau181, tau phosphorylated at threonine 181; p-tau202, tau phosphorylated at serine 202; p-tau205, tau phosphorylated at threonine 205; PET, positron emission tomography; SUVR, standardized uptake value ratio; t-tau, total tau, VBM, voxel based morphometry.

**Supplementary Table 4. Demographics of the 220 participants of the TRIAD cohort stratified using Aβ-PET and tau-PET into AT groups.**

| **AT PET (TRIAD cohort, n=220)** | **A-T-**  **(n=134)** | **A+T-**  **(n=43)** | **A+T+**  **(n=39)** | **A-T+**  **(n=4)** | ***P*-value** |
| --- | --- | --- | --- | --- | --- |
| **Age, years** | 59.97 (19.59) | 71.04 (6.58) | 66.11 (8.65) | 65.31 (9.01) | ˂0.001 |
| **Males (%)** | 58 (43.28) | 18 (41.86) | 18 (46.15) | 1 (25.0) | ns |
| **Level of education, years** | 15.29 (3.37) | 14.70 (3.18) | 15.39 (2.93) | 16.00 (5.89) | ns |
| **APOE-ε4 carriers (%)** | 33/133 (24.81) | 19/42 (45.24) | 24/36 (66.67) | 1/3 (33.33) | ˂0.001 |
| **MMSE score(available cases)** | 125 | 39 | 35 | 4 |  |
| **MMSE score** | 28.83 (2.44) | 28.21 (3.08) | 23.00 (6.38) | 21.50 (3.11) | ˂0.001 |
| **Lumipulse CSF (pg/mL)** |  |  |  |  |  |
| **Aβ42/40** | 0.09 (0.01) | 0.05 (0.01) | 0.04 (0.01) | 0.03 (0.01) | ˂0.001 |
| **p-tau181** | 32.62 (13.11) | 66.50 (33.86) | 106.33 (51.29) | 248.40 (140.93) | ˂0.001 |
| **t-tau** | 287.61 (150.52) | 488.98 (315.78) | 678.00 (311.07) | 1362.25 (685.38) | ˂0.001 |
| **Aβ-PET (available cases)** | 134 | 43 | 39 | 4 |  |
| **SUVR** | 1.28 (0.10) | 2.09 (0.42) | 2.57 (0.35) | 1.40 (0.14) | ˂0.001 |
| **Tau-PET (available cases)** | 134 | 43 | 39 | 4 |  |
| **SUVR** | 0.83 (0.09) | 0.96 (0.14) | 2.21 (0.74) | 3.18 (0.67) | ˂0.001 |
| **VBM (available cases)** | 126 | 42 | 36 | 4 |  |
| **mm3** | 0.47 (0.08) | 0.45 (0.06) | 0.40 (0.07) | 0.39 (0.04) | ˂0.01 |
| **GU CSF biomarkers (pg/mL)** |  |  |  |  |  |
| **p-tau202** | 1.70 (1.06) | 2.20 (0.99) | 3.27 (1.48) | 4.86 (1.75) | ˂0.001 |
| **p-tau205** | 1.69 (0.54) | 3.11 (1.44) | 5.03 (2.05) | 9.49 (4.88) | ˂0.001 |

Data is shown as mean (SD) or n (%), as appropriate. Kruskal Wallis test was used to compare age between groups and Pearson’s chi-square to compare sex and *APOE*-ε4 frequencies between groups. Years of education, MMSE and biomarkers levels were compared with a one-way ANOVA adjusted by age and sex. *Abbreviations*: Aβ42/40, ratio β-amyloid 42 and 40; A-T-, amyloid and tau negative; A+T-, amyloid positive tau negative; A+T+, amyloid and tau positive; A-T+, amyloid negative and tau positive; CSF, cerebrospinal fluid; GU, Gothenburg University Simoa assay; MMSE, Mini-Mental State Examination; ns, non-significant; p-tau181, tau phosphorylated at threonine 181; p-tau202, tau phosphorylated at serine 202; p-tau205, tau phosphorylated at threonine 205; PET, positron emission tomography; SUVR, standardized uptake value ratio; t-tau, total tau, VBM, voxel based morphometry.

**Supplementary Table 5. Demographics of the subset of TRIAD participants including Aβ-PET and Tau-PET.**

| **TRIAD, PET subset (n=227)^a^** | **Young**  **(n=27)** | **CU-**  **(n=67)** | **MCI-**  **(n=14)** | **NonAD- (n=20)** | **CU+**  **(n=32)** | **MCI+**  **(n=34)** | **AD**  **(n=32)** | ***P-*value** |
| --- | --- | --- | --- | --- | --- | --- | --- | --- |
| **Age, years** | 23.03(1.88) | 69.22(8.75) | 70.31(10.26) | 62.61(7.51) | 70.48(7.08) | 71.18(6.06) | 64.96(7.31) | ˂0.001 |
| **Males (%)** | 11(40.7) | 27(40.3) | 8(57.1) | 8(40.0) | 13(40.6) | 14(41.2) | 15(46.9) | ns |
| **Level of education, years** | 16.72(1.53) | 15.24(4.11) | 13.86(3.57) | 14.20(3.74) | 14.31(3.44) | 15.91(3.19) | 14.97(3.12) | ˂0.001 |
| ***APOE-*ε4 carriers (%)** | 6/27(22.2) | 16/67(23.9) | 2/14(14.3) | 3/19(15.8) | 13/32(40.6) | 19/31(61.3) | 20/30(66.7) | ˂0.001 |
| **MMSE score(available cases)** | 27 | 63 | 13 | 14 | 30 | 29 | 30 |  |
| **MMSE score** | 29.78(0.51) | 29.24(0.96) | 28.00(1.53) | 25.93(6.07) | 29.13(0.90) | 28.10(1.99) | 20.83(6.21) | ˂0.001 |
| **Lumipulse CSF (pg/mL)** |  |  |  |  |  |  |  |  |
| **Aβ42/40** | 0.09(0.01) | 0.09(0.01) | 0.09(0.02) | 0.09(0.01) | 0.05(0.01) | 0.05(0.01) | 0.04(0.01) | ˂0.001 |
| **p-tau181** | 22.47(7.14) | 33.65(11.09) | 41.66(12.43) | 28.40(8.89) | 58.33(32.93) | 79.97(38.52) | 123.56(83.58) | ˂0.001 |
| **t-tau** | 195.41(47.61) | 288.66(116.88) | 326.07(96.80) | 276.80(144.42) | 418.16(187.55) | 505.82(211.71) | 840.81(488.63) | ˂0.001 |
| **Aβ-PET (available cases)** | 27 | 67 | 14 | 20 | 32 | 34 | 32 |  |
| **SUVR** | 1.21(0.07) | 1.29(0.12) | 1.37(0.16) | 1.26(0.22) | 1.83(0.48) | 2.33(0.55) | 2.30(0.51) | ˂0.001 |
| **Tau-PET (available cases)^b^** | 26 | 67 | 14 | 19 | 31 | 33 | 32 |  |
| **SUVR** | 0.84(0.08) | 0.83(0.09) | 0.81(0.09) | 0.82(0.11) | 0.95(0.23) | 1.33(0.55) | 2.37(0.89) | ˂0.001 |
| **VBM (Available cases)** | 26 | 62 | 14 | 17 | 30 | 30 | 30 |  |
| **mm^3^** | 0.57(0.06) | 0.45(0.05) | 0.44(0.04) | 0.41(0.07) | 0.46(0.05) | 0.43(0.06) | 0.39(0.07) | ˂0.001 |
| **GU CSF biomarkers (pg/mL)** |  |  |  |  |  |  |  |  |
| **p-tau202** | 1.05(0.72) | 1.73(0.85) | 1.50(0.66) | 2.20(1.54) | 2.20(1.18) | 2.58(1.26) | 3.44(1.54) | ˂0.001 |
| **p-tau205** | 1.19(0.37) | 1.76(0.48) | 1.92(0.40) | 1.57(0.52) | 2.73(1.39) | 3.87(1.61) | 5.49(3.04) | ˂0.001 |

Data is shown as mean (SD) or n (%), as appropriate. ^a^ NonAD+ group only included one participant. ^b^ Tau-PET was available in 223 individuals. Kruskal Wallis test was used to compare age between groups and Pearson’s chi-square to compare sex and APOE ε4 frequencies between groups. Years of education, MMSE and biomarkers levels were compared with a one-way ANOVA adjusted by age and sex. *Abbreviations*: Aβ42/40, ratio β-amyloid 42 and 40; AD, Alzheimer’s disease; CSF, cerebrospinal fluid; CU, cognitively unimpaired; GU, Gothenburg University Simoa assay; MCI mild cognitive impairment; MMSE, Mini-Mental State Examination; NonAD, non-Alzheimer’s disease; ns, non-significant; p-tau181, tau phosphorylated at threonine 181; p-tau202, tau phosphorylated at serine 202; p-tau205, tau phosphorylated at threonine 205; PET, positron emission tomography; SUVR, standardized uptake value ratio; t-tau, total tau, VBM, voxel based morphometry.

**Supplementary Table 6. Demographics of the subset of TRIAD participants including CSF p-tau181, p-tau217 and p-tau231 measurements.**

| **TRIAD cohort subset (n=202)^a^** | **Young**  **(n=25)** | **CU-**  **(n=51)** | **MCI-**  **(n=12)** | **NonAD-**  **(n=14)** | **CU+**  **(n=26)** | **MCI+**  **(n=25)** | **AD**  **(n=23)** | ***P*-value** |
| --- | --- | --- | --- | --- | --- | --- | --- | --- |
| **Age, years** | 23.23 (1.81) | 68.29 (9.87) | 70.72 (11.10) | 62.91 (7.30) | 70.87 (7.41) | 71.61 (5.42) | 63.52 (6.68) | ˂0.001 |
| **Males (%)** | 10 (40.0) | 20 (39.22) | 7 (58.3) | 5 (35.71) | 11 (42.31) | 11 (44.0) | 14 (60.87) | ns |
| **Formal education, years** | 16.7 (1.56) | 15.4 (3.74) | 13.8 (3.72) | 13.7 (3.08) | 13.8 (3.17) | 14.7 (4.11) | 14.7 (3.37) | ˂0.05 |
| ***APOE* ε4 carriers (%)** | 5/25 (20.0) | 15/51 (29.41) | 2/12 (16.7) | 2/14 (14.29) | 9/26 (34.62) | 15/25 (60.0) | 16/23 (69.57) | ˂0.001 |
| **MMSE score (available cases)** | 25 | 48 | 11 | 10 | 24 | 22 | 22 |  |
| **MMSE score** | 29.80 (0.50) | 29.17 (0.95) | 27.82 (1.60) | 25.50 (6.95) | 29.04 (0.96) | 28.05 (2.01) | 20.23 (6.61) | ˂0.001 |
| **Aβ-PET (available cases)** | 25 | 50 | 12 | 12 | 26 | 25 | 23 |  |
| **SUVR** | 1.21 (0.07) | 1.30 (0.13) | 1.34 (0.10) | 1.21 (0.09) | 1.87 (0.51) | 2.34 (0.47) | 2.30 (0.47) | ˂0.001 |
| **Tau-PET (available cases)** | 25 | 51 | 12 | 13 | 26 | 25 | 23 |  |
| **SUVR** | 0.84 (0.08) | 0.84 (0.09) | 0.82 (0.10) | 0.79 (0.11) | 0.95 (0.24) | 1.35 (0.59) | 2.30 (0.76) | ˂0.001 |
| **VBM (Available cases)** | 24 | 50 | 12 | 13 | 25 | 25 | 22 |  |
| **mm^3^** | 0.57 (0.07) | 0.46 (0.05) | 0.44 (0.04) | 0.41 (0.08) | 0.46 (0.05) | 0.43 (0.06) | 0.40 (0.06) | ˂0.001 |
| **§CSF biomarkers (pg/mL)** |  |  |  |  |  |  |  |  |
| **Aβ42/40** | 0.09 (0.01) | 0.09 (0.01) | 0.09 (0.01) | 0.09 (0.01) | 0.05 (0.01) | 0.05 (0.01) | 0.04 (0.01) | ˂0.001 |
| **t-tau** | 194.68 (48.97) | 293.06 (109.26) | 309.50 (94.30) | 266.71 (168.75) | 412.73 (199.46) | 528.88 (189.64) | 664.87 (346.31) | ˂0.001 |
| **p-tau181** | 22.44 (7.17) | 34.06 (11.09) | 39.52 (11.79) | 25.79 (7.85) | 59.80 (35.84) | 84.30 (33.15) | 103.05 (62.50) | ˂0.001 |
| **p-tau202** | 0.98 (0.68) | 1.79 (0.86) | 1.46 (0.70) | 2.09 (1.35) | 2.06 (1.13) | 2.79 (1.27) | 3.19 (1.32) | ˂0.001 |
| **p-tau205** | 1.17 (0.37) | 1.83 (0.47) | 1.90 (0.40) | 1.56 (0.48) | 2.81 (1.52) | 4.02 (1.36) | 4.82 (2.36) | ˂0.001 |
| **p-tau217** | 3.13 (1.79) | 4.97 (3.11) | 5.31 (1.53) | 4.36 (2.39) | 15.13 (18.39) | 21.55 (10.75) | 29.79 (21.85) | ˂0.001 |
| **p-tau231** | 155.93 (83.51) | 252.31 (109.92) | 278.74 (118.26) | 193.04 (79.87) | 525.02 (440.87) | 690.60 (245.77) | 857.29 (562.43) | ˂0.001 |

Data is shown as mean (SD) or n (%), as appropriate. ^a^ NonAD+ group only included one participant. Kruskal Wallis test was used to compare age between groups and Pearson’s chi-square to compare sex and APOE ε4 frequencies between groups. Years of education, MMSE and biomarkers levels were compared with a one-way ANOVA adjusted by age and sex. *Abbreviations*: Aβ42/40, ratio β-amyloid 42 and 40; AD, Alzheimer’s disease; CSF, cerebrospinal fluid; CU, cognitively unimpaired; MCI mild cognitive impairment; MMSE, Mini-Mental State Examination; NonAD, non-Alzheimer’s disease; ns, non-significant; p-tau181, tau phosphorylated at threonine 181; p-tau202, tau phosphorylated at serine 202; p-tau205, tau phosphorylated at threonine 205; p-tau217, tau phosphorylated at threonine 217; p-tau231, tau phosphorylated at threonine 231; PET, positron emission tomography; SUVR, standardized uptake value ratio; t-tau, total tau, VBM, voxel based morphometry.

**Supplementary Table 7. Demographics of the subset of TRIAD participants including neurodegeneration measurements using MRI.**

| **VBM (n=213)^a^** | **Young (n=26)** | **CU-**  **(n=64)** | **MCI-**  **(n=14)** | **NonAD- (n=18)** | **CU+**  **(n=30)** | **MCI+**  **(n=30)** | **AD**  **(n=30)** | ***P-*value** |
| --- | --- | --- | --- | --- | --- | --- | --- | --- |
| **Age, years** | 23.07(1.91) | 69.22(9.53) | 70.31(10.26) | 63.75(8.42) | 70.54(6.86) | 71.38(5.57) | 64.91(7.31) | ˂0.001 |
| **Males (%)** | 11(42.3) | 25(39.1) | 8(57.1) | 5(27.8) | 12(40.0) | 13(43.3) | 14(46.7) | ns |
| **Level of education, years** | 16.79(1.52) | 15.70(3.87) | 13.86(3.57) | 14.11(3.31) | 14.33(3.52) | 15.53(2.69) | 15.07(3.19) | ˂0.001 |
| ***APOE-*ε4 carriers (%)** | 6/26(23.1) | 16/64(25.0) | 2/14(14.3) | 3/18(16.7) | 11/30(36.7) | 17/28(60.7) | 19/29(65.5) | ˂0.001 |
| **MMSE score(available cases)** | 26 | 62 | 13 | 14 | 29 | 27 | 28 |  |
| **MMSE score** | 29.77(0.51) | 29.26(0.96) | 28.00(1.53) | 25.93(6.07) | 29.10(0.90) | 28.00(2.02) | 21.50(4.97) | ˂0.001 |
| **Lumipulse CSF (pg/mL)** |  |  |  |  |  |  |  |  |
| **Aβ42/40** | 0.09(0.01) | 0.09(0.01) | 0.09(0.02) | 0.09(0.01) | 0.05(0.01) | 0.05(0.01) | 0.04(0.01) | ˂0.001 |
| **p-tau181** | 22.44(7.28) | 33.53(10.37) | 41.66(12.43) | 27.83(8.97) | 58.56(34.01) | 84.56(38.41) | 126.86(85.30) | ˂0.001 |
| **t-tau** | 196.42(48.26) | 284.81(102.54) | 326.07(96.80) | 279.78(149.57) | 422.47(193.12) | 527.27(214.44) | 863.03(496.48) | ˂0.001 |
| **Aβ-PET (available cases)** | 26 | 62 | 14 | 17 | 30 | 30 | 30 |  |
| **SUVR** | 1.21(0.07) | 1.30(0.13) | 1.37(0.16) | 1.27(0.23) | 1.82(0.47) | 2.37(0.50) | 2.27(0.51) | ˂0.001 |
| **Tau-PET (available cases)** | 25 | 64 | 14 | 18 | 29 | 30 | 30 |  |
| **SUVR** | 0.84(0.08) | 0.83(0.09) | 0.81(0.09) | 0.82(0.11) | 0.95(0.24) | 1.37(0.56) | 2.40(0.91) | ˂0.001 |
| **VBM (Available cases)** | 26 | 64 | 14 | 18 | 30 | 30 | 30 |  |
| **mm^3^** | 0.57(0.06) | 0.46(0.05) | 0.44(0.04) | 0.41(0.07) | 0.46(0.05) | 0.43(0.06) | 0.39(0.07) | ˂0.001 |
| **GU CSF biomarkers (pg/mL)** |  |  |  |  |  |  |  |  |
| **p-tau202** | 1.02(0.72) | 1.76(0.82) | 1.50(0.66) | 2.35(1.56) | 2.13(1.11) | 2.66(1.27) | 3.41(1.45) | ˂0.001 |
| **p-tau205** | 1.19(0.38) | 1.77(0.44) | 1.92(0.40) | 1.59(0.53) | 2.74(1.43) | 4.04(1.59) | 5.62(3.09) | ˂0.001 |

Data is shown as mean (SD) or n (%), as appropriate. ^a^ NonAD+ group only included one participant. Kruskal Wallis test was used to compare age between groups and Pearson’s chi-square to compare sex and APOE ε4 frequencies between groups. Years of education, MMSE and biomarkers levels were compared with a one-way ANOVA adjusted by age and sex. *Abbreviations*: Aβ42/40, ratio β-amyloid 42 and 40; AD, Alzheimer’s disease; CSF, cerebrospinal fluid; CU, cognitively unimpaired; GU, Gothenburg University Simoa assay; MCI mild cognitive impairment; MMSE, Mini-Mental State Examination; NonAD, non-Alzheimer’s disease; ns, non-significant; p-tau181, tau phosphorylated at threonine 181; p-tau202, tau phosphorylated at serine 202; p-tau205, tau phosphorylated at threonine 205; PET, positron emission tomography; SUVR, standardized uptake value ratio; t-tau, total tau, VBM, voxel based morphometry.

**2. SUPPLEMENTARY FIGURES**

**

**

**Supplementary Figure 1. Mass spectrometry validation of anti p-tau202 and p-tau205 antibodies.** The specificity of the two anti p-tau antibodies for their respective phosphorylated residue was evaluted through IP followed by LC-MS. Anti p-tau202 antibody exhibited a high-intensity peak solely with peptides containing phosphorylation at position 202 ([tau 195-209 p202] and [tau 190-214 p202]), while it did not react with peptides phosphorylated at position 205 ([tau 195-209 p205] and [tau 190-214 p205]) or the non-phosphorylated peptide ([tau195-209]). Similarly, anti-205 displayed a high-intensity peak only with peptides containing phosphorylation at position 205 ([tau 195-209 p205] and [tau 190-214 p205]), but did not react with peptides phosphorylated at position 202 ([tau 195-209 p202] and [tau 190-214 p202]) or the non-phosphorylated peptide ([tau195-209]).

**
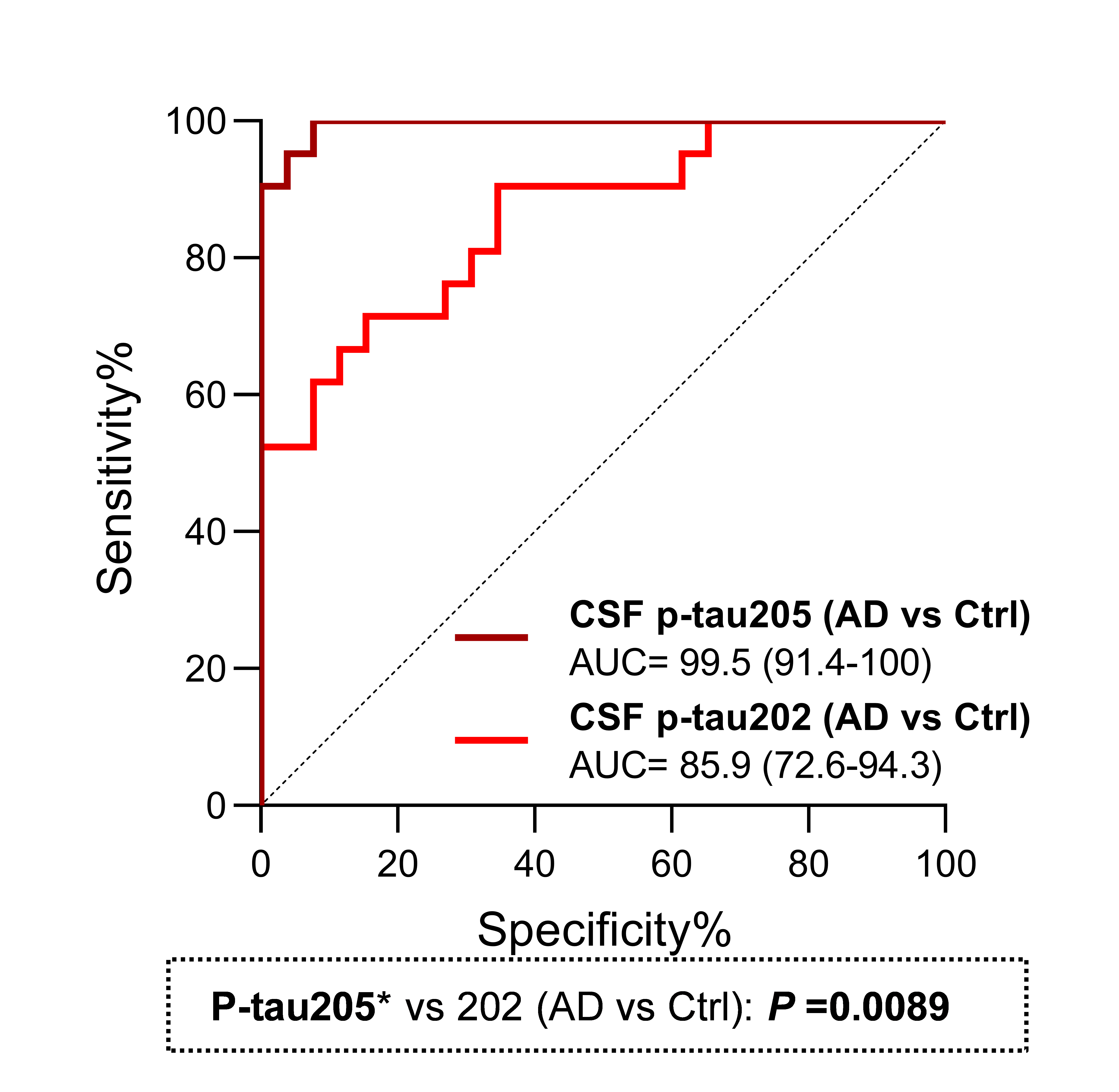
**

**Supplementary Figure 2. CSF p-tau205 and p-tau202 diagnostic performance in the Discovery cohort.** ROC analysis showing the higher diagnostic performance of CSF p-tau205 and p-tau202 when discriminating AD from control cases in the Discovery cohort. *Data information:* AUC values are presented followed by 95% confidence intervals. DeLong test (dashed square) was used to determine the statistical differences between biomarker performances (*P*˂0.05 is indicated in bold).


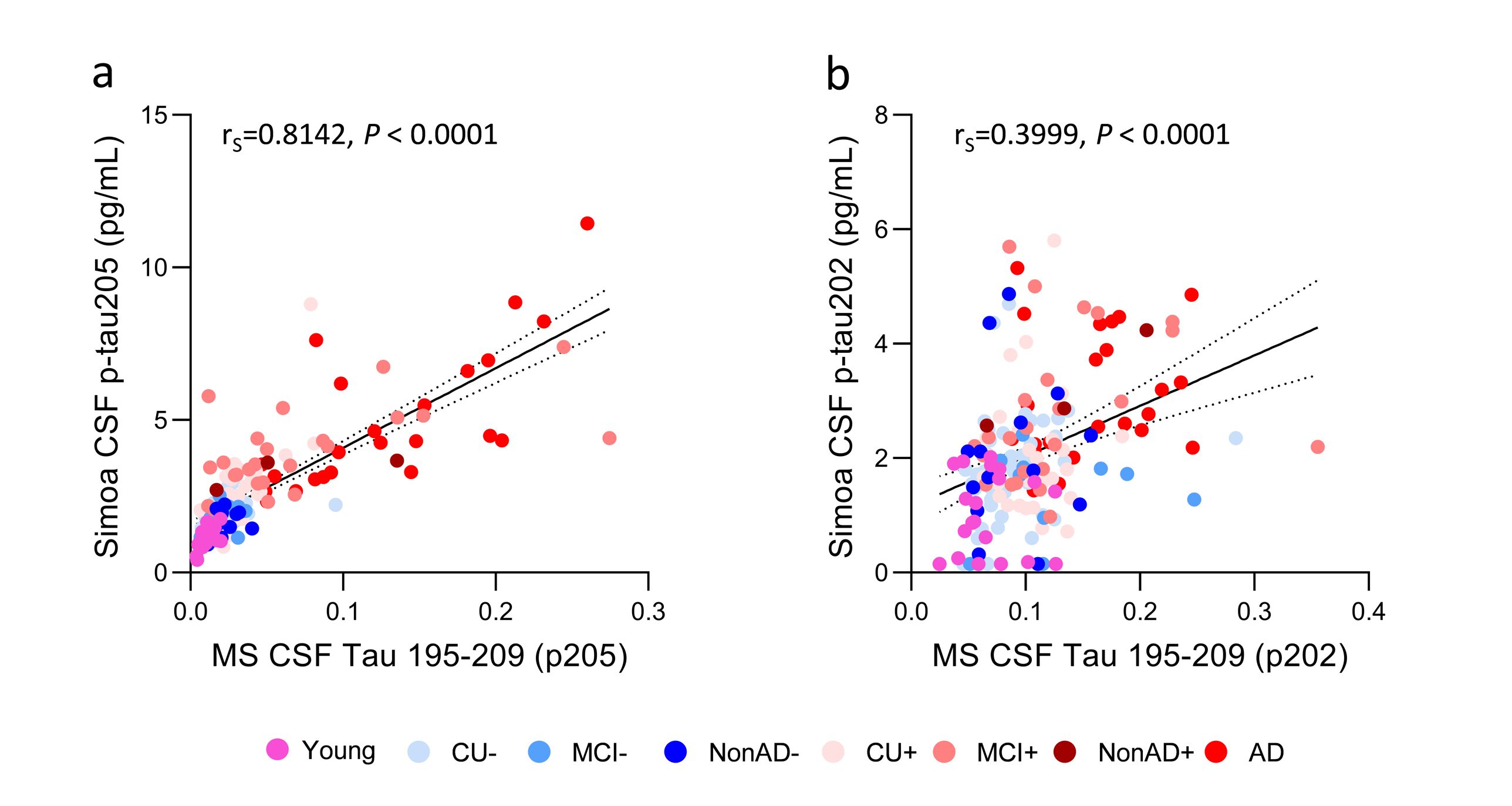


**Supplementary Figure 3. Spearman’s rank correlation between CSF p-tau205 and p-tau202 assays with antibody-free mass spectrometry in the TRIAD cohort. (a)** CSF p-tau205 and **(b)** CSF p-tau202 correlated with antibody-free mass spectrometry measurements of tryptic CSF p-tau205 and p-tau202 peptides in the TRIAD cohort. *Data information*: Participants colour-coded based on the presence (red tones) or absence (purple and blue tones) of CSF amyloidosis determined with Lumipulse CSF Aβ42/40. Spearman’s rank correlation and simple linear regression (with 95% confidence intervals) for all participants are displayed.





**Supplementary Figure 4. CSF p-tau205 and p-tau202 diagnostic performance discriminating diagnostic groups in the Paris cohort.** ROC analysis showing and comparing the performance of CSF p-tau205 and p-tau202 discriminating AD **(a, b)**, MCI+ **(c, d)** and nonAD+ **(e, f)** from CSF Aβ- diagnostic groups. *Data information:* AUC values are presented followed by 95% confidence intervals. DeLong test (dashed square) was used to determine the statistical differences between biomarker performances (*P*˂0.05 is indicated in bold).








**Supplementary Figure 5. CSF p-tau205 and p-tau202 diagnostic performance discriminating diagnostic groups in the TRIAD cohort.** ROC analysis showing and comparing the performance of CSF p-tau205 and p-tau202 discriminating AD **(a, b)**, MCI+ **(c, d)**, nonAD+ **(e, f)** and CU+ **(g, h)** from CSF Aβ- diagnostic groups. *Data information:* AUC values are presented followed by 95% confidence intervals. DeLong test (dashed square) was used to determine the statistical differences between biomarker performances (*P*˂0.05 is indicated in bold).





**Supplementary Figure 6. CSF p-tau205 and p-tau202 diagnostic performance discriminating AT groups in the Paris and TRIAD cohorts.** ROC analysis showing and comparing the performance of **(a)** CSF p-tau205 and **(b)** p-tau202 discriminating AT groups in the Paris cohort. ROC analysis showing and comparing the performance of **(c)** CSF p-tau205 and **(d)** p-tau202 discriminating AT groups in the TRIAD cohort. *Data information:* AUC values are presented followed by 95% confidence intervals. DeLong test (dashed square) was used to determine the statistical differences between biomarker performances (*P*˂0.05 is indicated in bold).





**Supplementary Figure 7. CSF p-tau205 and p-tau202 levels and performance across AT groups determined using Aβ and tau PET. (a)** CSF p-tau205 increased progressively across AT groups **(b)** whereas CSF p-tau202 was only increased in the A+T+ group. ROC analysis showing and comparing the performance of **(c)** CSF p-tau205 and **(d)** p-tau202 discriminating PET defined AT groups in the TRIAD cohort. Data information: Boxplots show the median, IQR and all participants. Participants colour-coded based on the presence (red) or absence (blue) of amyloidosis determined with Aβ PET. P-values were determined using one-way ANOVA adjusted by age and sex, followed by Bonferroni-corrected post hoc comparison (*P <0.05, **P <0.01, ***P <0.001, ****P <0.0001). AUC values are presented followed by 95% confidence intervals. DeLong test (dashed square) was used to determine the statistical differences between biomarker performances (*P*˂0.05 is indicated in bold).


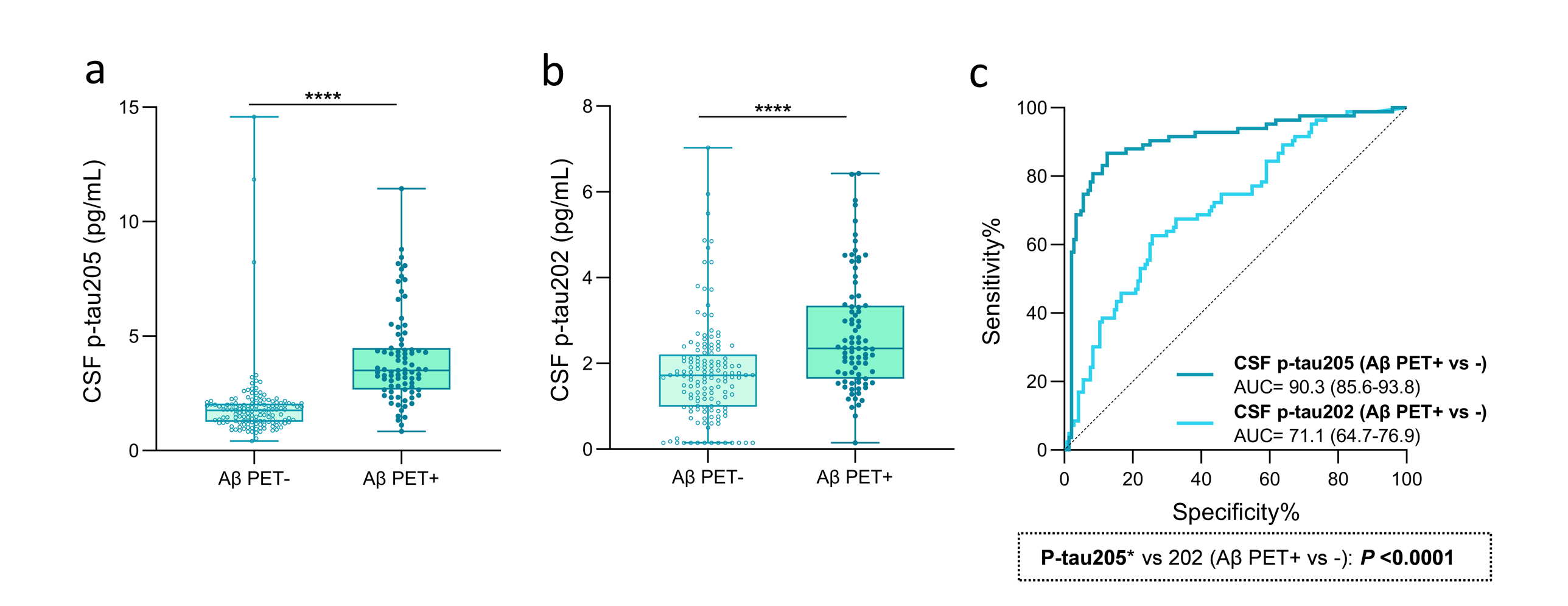


**Supplementary Figure 8. CSF p-tau205 and p-tau202 concentrations and diagnostic performance in in** **Aβ-PET negative and positive participants in the TRIAD cohort.** In the TRIAD cohort, both **(a)** CSF p-tau205 and **(b)** p-tau202 were increased in Aβ-PET positive compared with Aβ-PET negative cases. **(c)** ROC analysis showing the higher diagnostic performance (in AUC values) of CSF p-tau205 and p-tau202 when discriminating Aβ-PET positive from Aβ-PET negative cases. *Data information:* Boxplots show the median, IQR and all participants. Group differences were determined using Mann-Whitney *U* test (*****P* <0.0001). AUC values are presented followed by 95% confidence intervals. DeLong test (dashed square) was used to determine the statistical differences between biomarker performances (*P*˂0.05 is indicated in bold).





**Supplementary Figure 9. Spearman’s rank correlation between CSF p-tau205 and p-tau202 concentrations with tau-PET SUVRs across diagnostic groups in the TRIAD cohort. (a)** CSF p-tau205 levels correlated with tau-PET SUVRs across all CSF Aβ+ diagnostic groups (CU+, MCI+ and AD+). **(b)** CSF p-tau202 levels only correlated with tau-PET SUVRs across in AD cases. *Data information*: Participants colour-coded based on the presence (red tones) or absence (purple and blue tones) of CSF amyloidosis determined with Lumipulse CSF Aβ42/40. Spearman’s rank correlations of all diagnostic groups are displayed. Simple linear regressions (with 95% confidence intervals) of diagnostic groups which showed significant correlations with tau-PET SUVRs are presented.


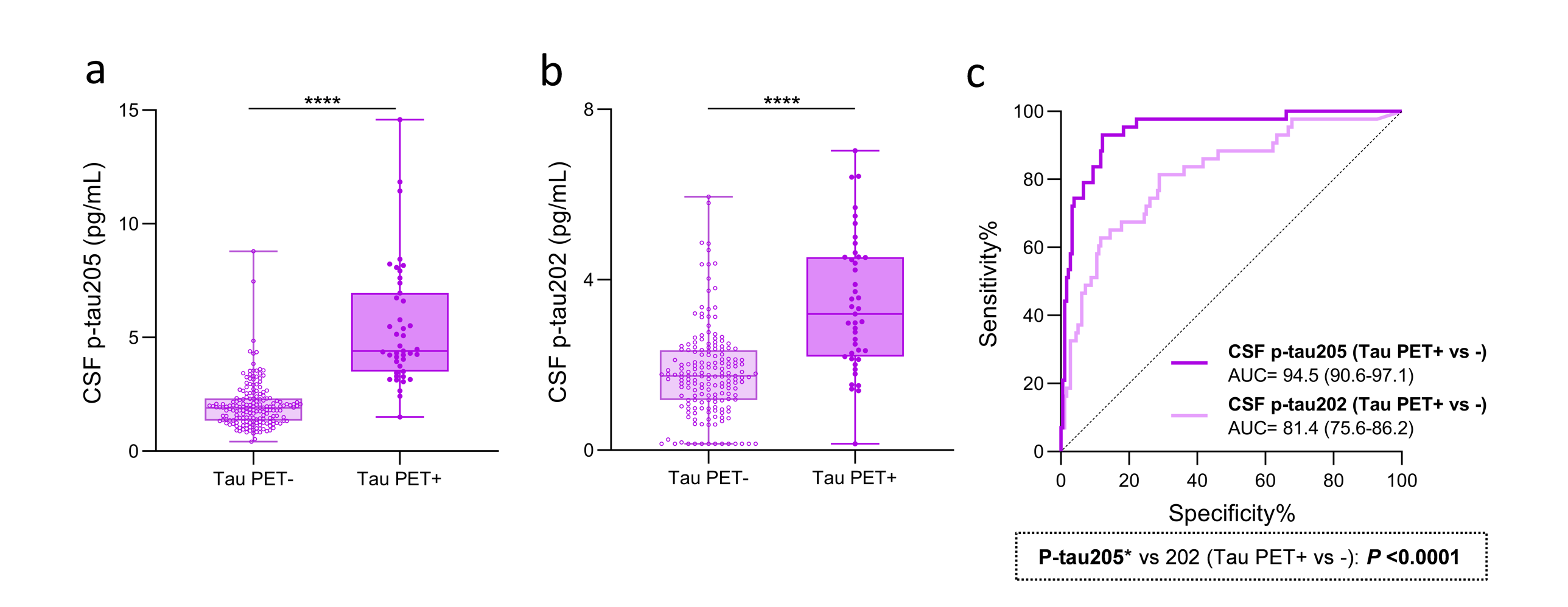


**Supplementary Figure 10. CSF p-tau205 and p-tau202 concentrations and diagnostic performance in in tau-PET negative and positive participants in the TRIAD cohort.** In the TRIAD cohort, both **(a)** CSF p-tau205 and **(b)** p-tau202 were increased in tau-PET positive compared with tau-PET negative cases. **(c)** ROC analysis showing the higher diagnostic performance (in AUC values) of CSF p-tau205 and p-tau202 when discriminating tau-PET positive from tau-PET negative cases. *Data information:* Boxplots show the median, IQR and all participants. Group differences were determined using Mann-Whitney *U* test (*****P* <0.0001). AUC values are presented followed by 95% confidence intervals. DeLong test (dashed square) was used to determine the statistical differences between biomarker performances (*P*˂0.05 is indicated in bold).





**Supplementary Figure 11.** **Proportion of variation in CSF p-tau biomarkers explained by Aβ and tau pathology measured by PET (TRIAD cohort).** The performance of three regression models (Aβ-PET: A, tau-PET: T, Aβ and tau-PET: A+T) predicting CSF p-tau181, p-tau202, p-tau205, p-tau217, and p-tau231 concentrations was assessed. The best-fitting model in all cases was A+T. CSF p-tau205 showed the highest R^2^ (A+T model: R^2^=0.636), with T accounting for the highest partial R^2^ = 0.32. *Data information:* Each bar plot represents one model. Independent variables included Aβ-PET (A, in red) and tau-PET (T in blue). All models include age and sex as covariates (represented in grey). AIC of each model is displayed on top of each bar plot, within a dashed square. R-squared values for each model are displayed on top of the respective bar plot, whereas the partial R-squared of each variable within the model is presented inside the bar plot.





**Supplementary Figure 12. CSF p-tau205 and p-tau202 associations with neurodegeneration. (a)** CSF p-tau205 correlated with global measures of grey matter atrophy assessed by voxel-based morphometry (VBM) across all cases and CSF Aβ+ and Aβ- participants. **(b)** CSF p-tau202 correlated with global measures of grey matter atrophy across all cases and CSF Aβ- participants. Both **(c)** CSF p-tau205 and (**d)** CSF p-tau202 displayed associations with grey matter loss also at voxel-level. *Data information*: Participants colour-coded based on the presence (red) or absence (blue) of CSF amyloidosis measured with Lumipulse CSF Aβ42/40. Spearman’s rank correlation is displayed for all participants, CSF Aβ+ and Aβ- groups. Simple linear regression with 95% confidence intervals of CSF Aβ+ and Aβ- groups is also presented. Voxel maps display the adjusted R-squared and t values of the linear associations between CSF biomarkers and VBM, adjusted by age and sex.

**REFERENCES**

1 Ashton NJ, Pascoal TA, Karikari TK, Benedet AL, Lantero-Rodriguez J, Brinkmalm G, Snellman A, Scholl M, Troakes C, Hye Aet al (2021) Plasma p-tau231: a new biomarker for incipient Alzheimer's disease pathology. Acta Neuropathol 141: 709-724 Doi 10.1007/s00401-021-02275-6

2 Brinkmalm G, Portelius E, Ohrfelt A, Mattsson N, Persson R, Gustavsson MK, Vite CH, Gobom J, Mansson JE, Nilsson Jet al (2012) An online nano-LC-ESI-FTICR-MS method for comprehensive characterization of endogenous fragments from amyloid beta and amyloid precursor protein in human and cat cerebrospinal fluid. J Mass Spectrom 47: 591-603 Doi 10.1002/jms.2987

3 Cicognola C, Brinkmalm G, Wahlgren J, Portelius E, Gobom J, Cullen NC, Hansson O, Parnetti L, Constantinescu R, Wildsmith Ket al (2019) Novel tau fragments in cerebrospinal fluid: relation to tangle pathology and cognitive decline in Alzheimer's disease. Acta Neuropathol 137: 279-296 Doi 10.1007/s00401-018-1948-2

4 Gobom J, Benedet AL, Mattsson-Carlgren N, Montoliu-Gaya L, Schultz N, Ashton NJ, Janelidze S, Servaes S, Sauer M, Pascoal TAet al (2022) Antibody-free measurement of cerebrospinal fluid tau phosphorylation across the Alzheimer's disease continuum. Mol Neurodegener 17: 81 Doi 10.1186/s13024-022-00586-0

5 Karikari TK, Emersic A, Vrillon A, Lantero-Rodriguez J, Ashton NJ, Kramberger MG, Dumurgier J, Hourregue C, Cucnik S, Brinkmalm Get al (2021) Head-to-head comparison of clinical performance of CSF phospho-tau T181 and T217 biomarkers for Alzheimer's disease diagnosis. Alzheimers Dement 17: 755-767 Doi 10.1002/alz.12236

6 Karikari TK, Pascoal TA, Ashton NJ, Janelidze S, Benedet AL, Rodriguez JL, Chamoun M, Savard M, Kang MS, Therriault Jet al (2020) Blood phosphorylated tau 181 as a biomarker for Alzheimer's disease: a diagnostic performance and prediction modelling study using data from four prospective cohorts. Lancet Neurol 19: 422-433 Doi 10.1016/S1474-4422(20)30071-5

7 Lantero-Rodriguez J, Snellman A, Benedet AL, Mila-Aloma M, Camporesi E, Montoliu-Gaya L, Ashton NJ, Vrillon A, Karikari TK, Gispert JDet al (2021) P-tau235: a novel biomarker for staging preclinical Alzheimer's disease. EMBO Mol Med 13: e15098 Doi 10.15252/emmm.202115098

8 Pino LK, Searle BC, Bollinger JG, Nunn B, MacLean B, MacCoss MJ (2020) The Skyline ecosystem: Informatics for quantitative mass spectrometry proteomics. Mass Spectrom Rev 39: 229-244 Doi 10.1002/mas.21540
